# X-ray crystallographic characterization of the SARS-CoV-2 main protease polyprotein cleavage sites essential for viral processing and maturation

**DOI:** 10.1038/s41467-022-32854-4

**Published:** 2022-09-03

**Authors:** Jaeyong Lee, Calem Kenward, Liam J. Worrall, Marija Vuckovic, Francesco Gentile, Anh-Tien Ton, Myles Ng, Artem Cherkasov, Natalie C. J. Strynadka, Mark Paetzel

**Affiliations:** 1 Department of Biochemistry and Molecular Biology and Centre for Blood Research, The University of British Columbia, Vancouver, BC V6T 1Z3 Canada; 2 Department of Molecular Biology and Biochemistry, Simon Fraser University, Burnaby, BC V5A 1S6 Canada; 3 Vancouver Prostate Centre, The University of British Columbia, Vancouver, BC V6H 3Z6 Canada

**Keywords:** X-ray crystallography, Enzyme mechanisms, SARS-CoV-2

## Abstract

Severe Acute Respiratory Syndrome Coronavirus 2 (SARS-CoV-2), the pathogen that causes COVID-19, produces polyproteins 1a and 1ab that contain, respectively, 11 or 16 non-structural proteins (nsp). Nsp5 is the main protease (M^pro^) responsible for cleavage at eleven positions along these polyproteins, including at its own N- and C-terminal boundaries, representing essential processing events for viral assembly and maturation. Using C-terminally substituted M^pro^ chimeras, we have determined X-ray crystallographic structures of M^pro^ in complex with 10 of its 11 viral cleavage sites, bound at full occupancy intermolecularly in trans, within the active site of either the native enzyme and/or a catalytic mutant (C145A). Capture of both acyl-enzyme intermediate and product-like complex forms of a P2(Leu) substrate in the native active site provides direct comparative characterization of these mechanistic steps as well as further informs the basis for enhanced product release of M^pro^’s own unique C-terminal P2(Phe) cleavage site to prevent autoinhibition. We characterize the underlying noncovalent interactions governing binding and specificity for this diverse set of substrates, showing remarkable plasticity for subsites beyond the anchoring P1(Gln)-P2(Leu/Val/Phe), representing together a near complete analysis of a multiprocessing viral protease. Collectively, these crystallographic snapshots provide valuable mechanistic and structural insights for antiviral therapeutic development.

## Abstract (web summary)

The SARS-CoV-2 protease Mpro is essential for viral replication. Here, the authors have determined the structures of Mpro in complex with 10 of the 11 viral cleavage sequences including a covalent acyl-enzyme intermediate, providing mechanistic and structural insights for antiviral development.

## Introduction

**Fig. 1:**
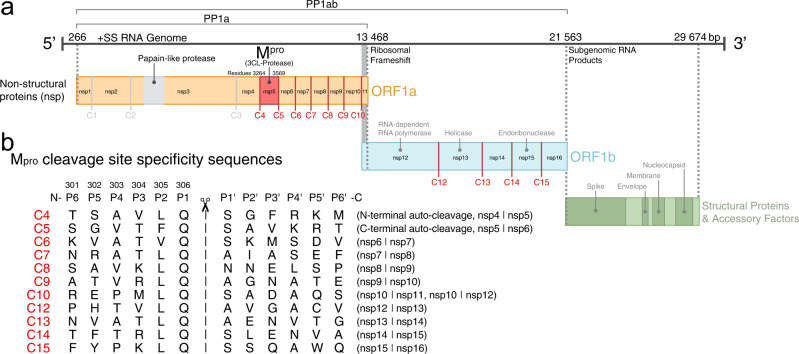
M^pro^ cleavage sites in SARS-CoV-2 polyprotein processing. **a** Schematic overview of the SARS-CoV-2 genome. Open reading frames (ORF) 1a and 1b encode polyproteins pp1a and pp1ab that together contain a total of 11 M^pro^ cleavage sites (C4-10, C12-C15, red). **b** Sequence alignment of the SARS-CoV-2 M^pro^ cleavage-site specificity residues (P6–P6′). Amino acid numbering above P6–P1 is as for C5, the C-terminal autocleavage sequence of M^pro^ itself, the template into which all other cleavage site P6–P1 sequences were systematically substituted in this study.

The SARS-CoV-2 genome encodes four structural proteins and two overlapping polyproteins, pp1a and pp1ab, encompassing all the viral proteins required for host invasion and maintenance of the viral lifecycle (Fig. 1)^1,2^. These long polyproteins are processed into 16 smaller functional non-structural proteins (nsps) by two self-encoded cysteine proteases, papain-like protease (nsp3) and the main protease (nsp5). The main protease of SARS-CoV-2 (M^pro^, also referred to as 3-chymotrypsin-like protease or 3CLpro) is responsible for the majority of nsp processing, cleaving at 11 conserved sites along the polyprotein including self-excision of M^pro^ by autolytic cleavage of its own N-terminal and C-terminal autoprocessing sites^3^. These processing events by M^pro^ represent critical steps prior to subsequent viral assembly and maturation^3^. The functional importance of M^pro^ in the viral life cycle, combined with the absence of closely related homologs in humans and high degree of conservation of M^pro^ and its targets among clinical variants (~96% identical to SARS-CoV-1 M^pro^)^4^, has made the enzyme an attractive target for the development of antiviral drugs. This is highlighted by the recent success of nirmatrelvir (Paxlovid) in mitigation of serious COVID-19 disease and hospitalization in high-risk patients^5^. In addition, increasing evidence shows host cell protein cleavage by viral proteases is a further critical component of viral pathogenicity^6^ and recent proteomic analyses have revealed more than 100 substrates cleaved by M^pro^ in human lung and kidney cells including key effectors of transcription, mRNA processing, and translation^7,8^.

SARS-CoV-2 M^pro^ is a functional homodimer of two symmetrically disposed protomers that are each 306 residues in length (33.8 kDa). Each protomer is composed of three domains^9,10^; the first two are antiparallel β-barrel structures (residues 8–101 and 102–184) which together form the substrate binding pocket, and the third is an α-helical domain (residues 201–306) contributing to the extended dimerization interface essential for enzyme activity^11–14^. The substrate binding cleft of M^pro^ in SARS-CoV-2 and other coronaviruses is known to be permissive^15^, accommodating the binding of a diverse combination of residues within the substrate specificity binding pockets flanking the scissile bond (Fig. 1). Amongst the 11 cleavage sites of pp1a and pp1ab, there is little sequence conservation beyond an absolutely conserved glutamine in P1, hydrophobic (leucine predominant) at P2, and restriction to a small, predominantly aliphatic P4 and either a serine, alanine or asparagine in P1′. The active site of M^pro^ features a Cys145-His41 catalytic dyad^16^, which orchestrates nucleophilic attack at the carbonyl carbon of the invariant P1 glutamine and the formation of an acyl enzyme intermediate central to efficient peptide cleavage.

SARS-CoV-2 M^pro^ continues to be heavily studied, with crystallographic structures of the protease in native forms^8,13,15^ and with various bound chemical fragments and inhibitors reported^9,10,17–19^. Structures of catalytically inactive M^pro^ mutants in complex with peptides corresponding to different pp1ab cleavage sites are also more recently becoming available, providing valuable information on substrate specificity and the impact on drug design and potential emergence of drug resistance^20–23^. Previously, we described high-resolution structures of M^pro^ in complex with the native C-terminal autocleavage sequence of a symmetry neighbor in the crystal, capturing both product and acyl-enzyme intermediate states^24^ (cleavage site C5 in the nomenclature used in this paper; Fig. 1). Capitalizing on this approach, here we present high-resolution crystallographic structures of M^pro^ in complex with 10 of the 11 target polyprotein cleavage sequences, adding to the repertoire of defined M^pro^ cut site binding, yielding a consistent set for all but one of the 11. These complexes provide valuable snapshots into the molecular details governing substrate recognition and cleavage by M^pro^, allowing for structural insights for ongoing antiviral therapeutic development. As well we believe the work provides an interesting complement to prior peptide structures given we have captured the product mimics here using protein tethered peptides perhaps more in keeping with the physiological restraints in the intact polyprotein substrate, providing high effective concentration and clear density through P6.

## Results and discussion

### Production of SARS-CoV-2 C-terminal M^pro^ functional chimeras

Through extensive screening of various crystallization conditions/methods and X-ray crystallographic analyses, we previously were able to trap the intermolecular interaction of the C-terminal M^pro^ P6–P1 autocleavage sequence (C5) bound at full occupancy within the active site of a neighboring protomer in the crystal, likely afforded by the effective local concentration inherent to crystallization. This was achieved for both the native protein, which formed a covalent acyl-enzyme intermediate with the catalytic cysteine 145, and an inactivated C145A catalytic mutant which enabled the capture of a P6–P1 product-like complex of the bound C-terminal sequence^24^ (PDB 7KHP and 7JOY respectively). Here, we capitalize on this approach to characterize the other 10 equivalent non-prime side cleavage sequences (P6–P1, the main determinants of specificity^25^) along the SARS-CoV-2 polyprotein precursor pp1ab. To do this we have created chimeric M^pro^ variants in which we have systematically substituted the C-terminal 6 residues of M^pro^ (301–306) to each respective P6–P1 cleavage sequence (for brevity in text and labeling of figures denoted here as C4 through C10, C12 through C15; see Fig. 1 for definition of cleavage site nomenclature). This was performed in context of both the WT SARS-CoV-2 M^pro^ active site and C145A catalytic mutant backgrounds (see Supplementary Table 1 for complete list of primers). The constructs were cloned into a modified pET-28a plasmid including a N-terminal 6xHis-tag followed by a protease cleavable SUMO tag, resulting in tagless M^pro^ (Ser1-Gln306) variants purified to >95% homogeneity using standard chromatographic approaches (see “Methods”). Interestingly, the only variant not to be captured in our structures, C15, was also the most poorly behaved, readily precipitating out of solution suggestive of a more conformationally labile nature.

### Crystallographic analysis of SARS-CoV-2 polyprotein P6–P1 variants bound within the active site of M^pro^

Using the above purified chimeric variants, high throughput screening of thousands of conditions resulted in >500 hundred crystal hits, synchrotron data for which were subsequently collected, processed and analyzed for those with productive intermolecular complexes of enzyme/substrate interactions (see “Methods” and Supplementary Tables 2, 3 and 4 for crystallization conditions, crystallographic and model statistics, and refinement approaches, respectively). In this way, we have successfully determined product-like structures for all but one M^pro^ cleavage sequence (C15) bound in the active site of the C145A catalytic mutant, and two in complex with the native active site, one of which has formed an acyl-enzyme intermediate (see Supplementary Fig. 1 for representative electron density maps of bound cleavage sites with final refined models). In our previous structure of the autocatalytic C5 cut site^24^, we observed the C-terminal six residues had changed orientation from their more common location at the M^pro^ dimeric interface, instead extending across the C-terminal domain and binding into a neighboring active site of one protomer of an independent dimer in the crystal. We speculate this is likely occurring in crystallo, with favorable crystal packing of these “hit” complexes permitting the needed close association of the C-terminal region and neighboring active site. We clarify we are using the term “product” here to refer to the binding of the P6–P1 non-prime side residues that have been systematically introduced into the C-terminal region of the M^pro^ chimeras, mimicking the binding of the resulting product sequences from the M^pro^ catalyzed processing of pp1ab. These product mimics have been captured in either or both of the C145A and wild-type active sites at full occupancy by taking advantage of the high effective concentration of the in crystallo chimeric approach. We note the vast majority of crystals screened exhibited crystal packing that was non-productive of this specific interaction (empty active site), and in each case inspection of the electron density was ultimately necessary to identify the hits from the misses given the common crystal parameters and habits (<~10% of crystal data sets processed were productive complexes).

**Fig. 2:**
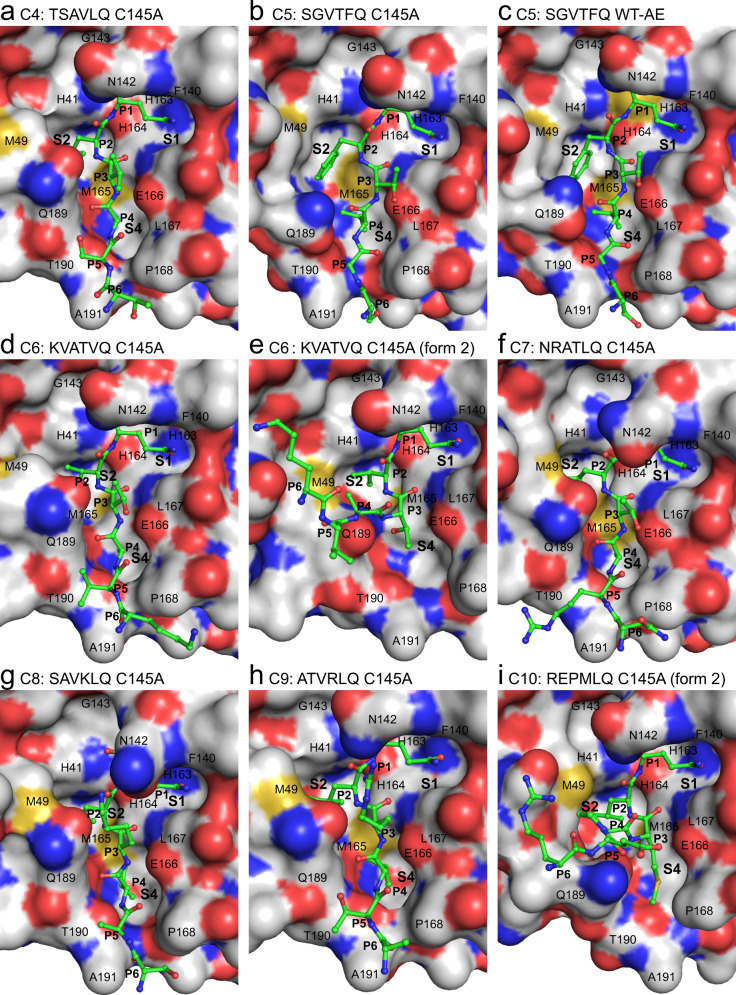
X-ray crystallographic structures of SARS-CoV-2 M^pro^ cleavage sites C4 to C10. **a**, **b** and **d**–**i** show product-like complexes with the C145A mutant, **c** shows an acyl-enzyme (AE) intermediate complex with wild-type (WT) M^pro^. The complementary substrate specificity residues (P6-P1) are labeled and shown as ball-and-stick (carbons are green) bound into the M^pro^ substrate binding groove shown as a molecular surface (carbons are gray). The Schechter–Berger substrate specificity pockets (S1, S2, and S4 in M^pro^) are labeled. Enzyme residues near the cleavage site atoms are labeled on the molecular surface. Non-carbon atoms are colored as follows: oxygen - red, nitrogen - blue, sulfur - yellow. The C5 C145A (**b**) and WT (**c**) structures were determined previously^24^ with PDB 7JOY and 7KHP, respectively, used to make the figure.

**Fig. 3:**
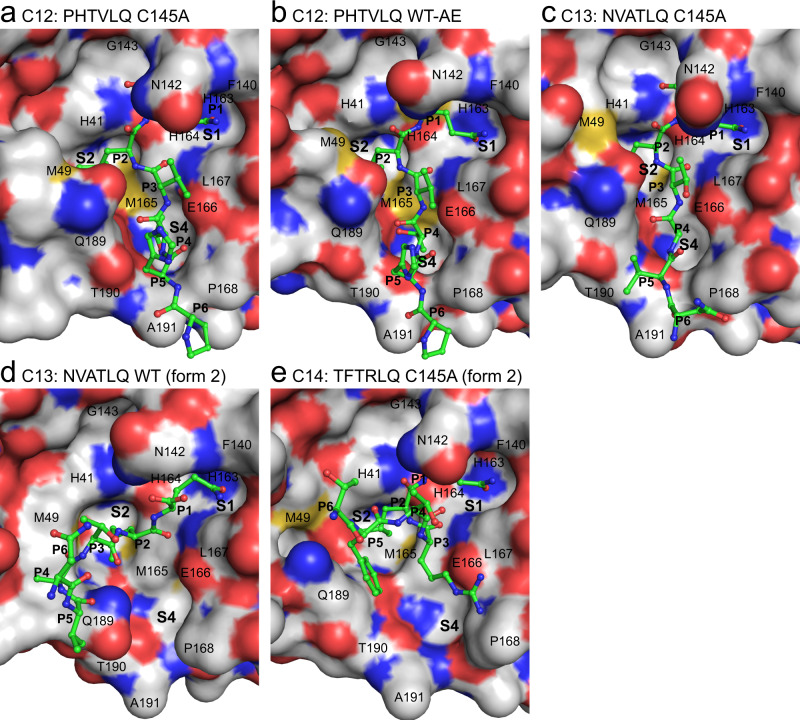
X-ray crystallographic structures of SARS-CoV-2 M^pro^ cleavage sites C12 to C14. **a** and **c**–**e** show product-like complexes with the C145A mutant, **b** shows an acyl-enzyme (AE) intermediate complex with wild-type (WT) M^pro^. Labels and colors as in Fig. 2.

We observe distinct crystal packing architectures (described by four space groups and eight unit cells) that permit the formation of the active site product and acyl enzyme complexes (illustrated in Supplementary Fig. 2). Cleavage site C8 has crystallized with the same approximate unit cell dimensions and relative M^pro^ dimer orientation in the crystal lattice as that of the earlier structure for C5; however, the C8 crystallographic data is defined by the space group P2_1_2_1_2_1_ with four protomer molecules in the asymmetric unit (ASU) compared to space group C2 and two molecules for C5. C4, C6 (form 1 - see below for definition; Figs. 2, 3, Supplementary Fig. 2, and Supplementary Tables 3, 5) and C12 also share a general relative disposition of M^pro^ dimers in the crystal packing but C4 and C6 have higher symmetry belonging to space group P22_1_2_1_ with three molecules in the ASU with the symmetry generated dimer contributing its C-terminus to one of the active sites of the non-crystallographic dimer. For cleavage site C12, in which we have captured complexes within both native and C145A active sites, crystals belong to space group P2_1_ with 12 protomers in the ASU, four of which create dimers with bound active site complexes. C6 (form 2), C10, C14, and C13WT have also crystallized in space group P2_1_ but with a distinct unit cell, two molecules in the ASU, and a unique conformation of the donor substrate (we term here form 2 - see also below; Figs. 2, 3, Supplementary Fig. 2 and Supplementary Tables 3 and 5). Finally, C7, C9, and C13 (all in the context of C145A) have crystallized as rings composed of seven dimers (Supplementary Fig. 2). These all belong to space group C2 with C7 and C13 having seven monomers (half a ring) in the ASU and C9 (C145A) having 14 (complete ring).

These fascinating, and in some instances extensive intermolecular formations observed in the crystal packing lead to questions as to the possible physiological relevance of these assemblies in regulation or stabilization in vivo as suggested in an earlier SARS-CoV-1 study^26^. Notably, the large 14-mer ring structure composed of seven dimers has been observed from three distinct C-terminal variants crystallizing in different conditions. Analysis of the crystal packing with PISA^27^ shows the main interface between dimers ranges in surface area from 800 to 1000 Å^2^ with the ring assembly as a whole predicted to be stable. However, we do not observe evidence of these in solution and oligomerization appears to occlude free access to the active sites for external substrates suggesting that barring some negative regulatory role or inactive storage form, the oligomers observed are likely crystallographic rather than physiological interactions.

### Analysis of the angle of approach and conformation of SARS-CoV-2 M^pro^ P6-P1 variants

In line with the significant variation in crystal space supporting productive complexes as above, significant variation in general approach of the protomers involved is also observed. Specifically, the substrate at the C-terminus of M^pro^ (donor) approaches the substrate binding groove within the corresponding M^pro^ (acceptor) at different angles, as defined here by the center of mass (donor) to P1 (Gln306Cα) (donor) to center of mass (acceptor) (Supplementary Fig. 2). The resulting observed angles can be placed into four generalized groups. We note that the crystallization conditions including pH and salt concentration were variable across all four groups, with no significant correlation evident that may have influenced the alternate binding modes (Supplementary Table 2). C4, C6 (form 1), C12 (WT and C145A) group together (Supplementary Fig. 2, green), with C8 grouped with the earlier C5 structures^24^ (Supplementary Fig. 2, yellow), all generally oriented with the donor chain C-terminal helical domain abutting the N-terminal β-barrel domain of the acceptor chain but at distinct angular dispositions. In the remaining structures, the donor chain is flipped approximately 180^o^ but again divided into two groups based on distinct angular dispositions as defined above (Supplementary Fig. 2, blue and red).

**Fig. 4:**
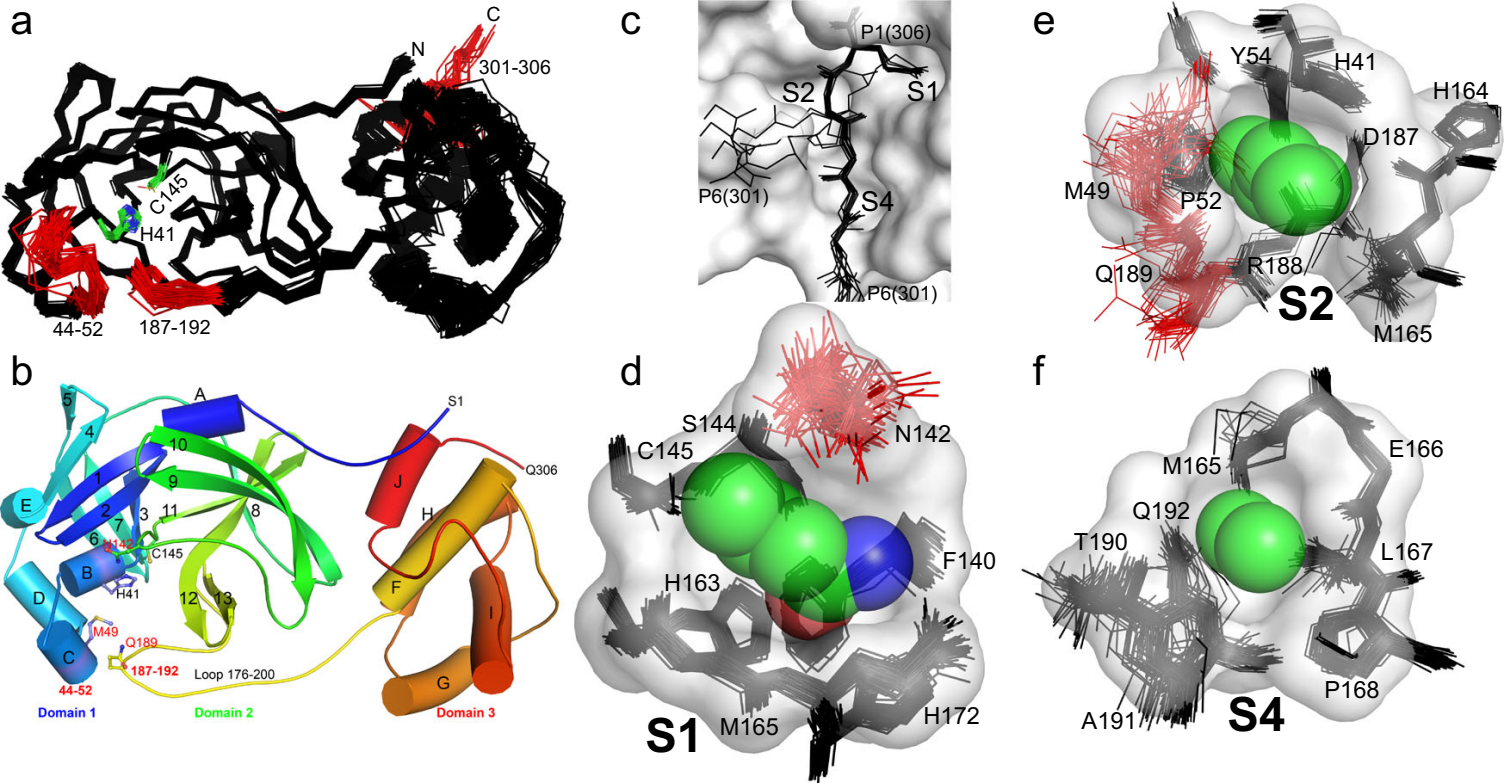
Plasticity of SARS-CoV-2 M^pro^ to promote molecular recognition of the polyprotein cleavage site variants. **a** Global structural alignment of all unique chains from the multiple structures characterized in this study. Structural alignments were performed using the ALIGN function in PyMOL with all protein atoms. Mobile regions are highlighted in red and labeled. Catalytic dyad H41/C145 are shown with green carbons and labeled. **b** M^pro^ cartoon highlighting secondary structural features, oriented as in panel **a**, colored spectrally - blue N-terminus to red C-terminus. **c** Overlay of representative P6 to P1 regions observed amongst the distinct cleavage site structures, highlighting their varying main chain conformations. The majority adopt the canonical extended β-type conformation within the binding site groove (see also Figs. 2 and 3), but four diverge (form 2); despite this the P1 Gln306 is remarkably fixed in position (side chain for Gln306 shown). **d** Structural alignment of all 74 S1 binding sites (stick) projected behind the S1 pocket (P1-Gln space filling and pocket surface is that of C4, provided for context). The P1 residue (Gln306) sidechain atoms are shown as semitransparent spheres (carbon - green, nitrogen - blue, oxygen - red). **e** Analogous all structure alignment of the S2 binding sites with space filling P2 (Leu305) and surface for the S2 pocket of C4. **f** Analogous all structure alignment of the S4 binding sites with space filling P4 (Ala303) and surface for the S4 pocket of C4.

Superposition of all M^pro^ chains in this study (Fig. 4a) shows marked conformational differences for not only the C-terminal P6–P1 residues 301–306, but as well the small largely 3_10_ helical region (residues 44–52) and a turn (residues 187–192) contained within a larger more ordered region (residues 176–200) that connects the catalytic and helical domain (Fig. 4a, b). The 3_10_ and turn regions abut the active site forming collectively the dynamic face of the cleft and contributing to all substrate specificity pockets, except, notably that for the anchoring P1(Gln) (see below). The plasticity and largely neutral/non-polar nature of these regions facing the active site (including sides chains of Met49, Gln189, and Gln192) accommodate a variety of non-covalent interactions for the varied P6–P2 orientations of donor observed (see details for each subsite below). Beyond P6, additional if subtle interactions of donor and acceptor are realized and vary with the particular approach in each case. Supplementary Fig. 3 provides a summary of representative donor/acceptor approach interfaces (P6–P1 excluded) and noncovalent interactions therein (approach groups defined and colored as per Supplementary Fig. 2). This summary shows 1-3% of the acceptor surface makes contact with the donor, and again a remarkable variety of positionings and contacts on either side of the active site cleft. Interestingly, a series of small predominantly aliphatic, fixed, and largely conserved side chains (Leu50, Ala191, Ala193, Pro168, Thr196, and Thr169), are positioned at the mouth of the binding groove, suggestive of a possible role in maintaining an open and neutral nature of the binding groove entrance proper (Supplementary Fig. 3). Although clearly our observations are based on chimeras of the same donor protein, these adaptable physicochemical features of the acceptor surface may facilitate the approach and binding of the diverse protein donors along the pp1ab polyprotein precursor and likely plays a similar role in accommodating the extensive potential substrates beyond that of virus^7,8^. Examination of experimentally determined (PDB 7CYQ, 6W4B, 6W4H, 6ZSL, and 6VWW) or AlphaFold^28^ predicted models suggest the C-terminally disposed cleavage sites in each of the pp1ab donor substrates are for the most part presented in a similarly tight fashion from a structured C-terminal element (several involving helices as for M^pro^), with few significant unstructured regions observed or predicted. Interestingly, an apparent exception is the predicted unstructured region between nsp7/nsp8 (C7), the cleavage site previously shown to have poorest cleavage efficiency amongst the M^pro^ substrates so far probed in the context of a polyprotein fragment^29^.

**Fig. 5:**
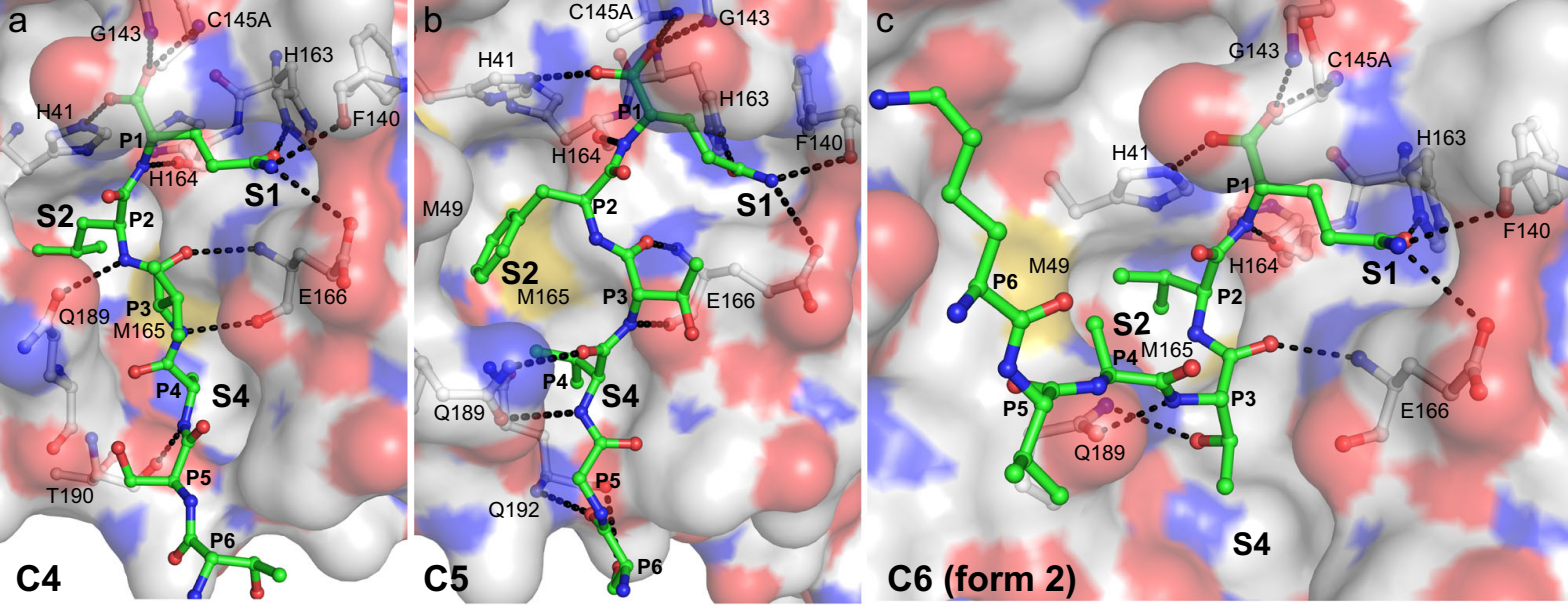
S2 specificity pocket rearrangements and hydrogen bond comparison of SARS-CoV-2 M^pro^ cleavage sites C4, C5, and C6. **a** Cleavage site C4 with P2(Leu). **b** Cleavage site C5 with P2(Phe) (PDB 7JOY^24^ used to make figure). **c** Cleavage site C6 form 2 with P2(Val). P6–P1 are shown in ball and stick with CPK coloring (carbons green) overlaid on the corresponding acceptor molecular surface with CPK coloring (carbons in light gray). Hydrogen bonds are shown as black dashed lines with donor and acceptor atoms labeled. Note repositioning of Gln189 and Met49 in particular.

A hallmark of proteases is an active site groove and underlying secondary structural features that facilitates binding of an extended, β-strand conformation of substrate, a canonical conformation that allows access by catalytic groups on both faces (*si* and *re*) to the scissile bond during cleavage^30^. However, our analysis of the main chain phi/psi conformations and overall path the substrates adopt within the acceptor active site sheds light on the adaptability of M^pro^ for binding of both canonical and non-canonical forms of the cleavage sites (Figs. 2, 3 and Supplementary Fig. 4). Superpositions of the representative P6-P1 cleavage regions captured here are provided in Fig. 4c with Supplementary Fig. 4 illustrating overlap of all occurrences of each cleavage site. These support the expected extended β conformation, we term here form 1, within the substrate binding groove in the majority of cleavage sites (C4, C5, C6-form1, C7, C8, C9, C12, and C13-form1), facilitating mainchain hydrogen bonding interactions from substrate with mainchain atoms of residues that make up the binding groove (His164 O, Glu166 N, O, and Thr190 O; Fig. 5). Interestingly, the others diverge from this canonical binding, we term here form 2: in C6 (form 2), C10, and C14 (crystallized isomorphously in space group P2_1_ with two molecules in the ASU) the substrate sequence is kinked ~ 90° at the P3 residues and tracks instead between the dynamic Met49 and Gln189 regions (Figs. 2e, i, 3d, e, 4c, and 5c). This altered substrate trajectory appears to be coincident with the close contact of the donor C-terminal domain in this crystal packing. Regardless of this non-canonical binding conformation, the vdW and hydrogen bond interactions of the P1 (Gln306) side chain and binding of the hydrophobic P2 residue in the S2 subsite are largely preserved (Figs. 4c, 5 and Supplementary Fig. 5) reinforcing the importance of these substrate positions in anchoring the needed conformation of the productive complex with more plasticity allowed as one moves away from the scissile bond (discussed further below).

### Analysis of the non-covalent interactions underlying M^pro^ substrate specificity

The ensemble of full occupancy structures captured in this study highlights the importance of highly conserved positions in the cleavage sequence as well as accommodation of relatively sequence diverse positions in M^pro^ substrate recognition and specificity. A molecular surface view of the binding modes of each of the P6–P1 variants across the catalytic cleft is illustrated in Figs. 2 and 3. The underlying hydrogen bonds observed for the main chain and side atoms of these complexes are also provided in Fig. 5 and Supplementary Fig. 5 with standard deviations and ranges listed in Supplementary Table 7 for the common interactions across multiple structures. Measurement of the buried surface area of individual substrate P residues within the active site is provided in Supplementary Table 6.

#### P1 position

Moving from the scissile bond position down through P6, from our analysis it is clear that by far the most conserved position in type and ES interaction profile is the P1 glutamine, the apparent binding crux of the diverse M^pro^ viral substrates regardless of their differing angles of approach or conformation as described above. Gln306 is highly complementary to the S1 enzyme pocket that accommodates it, formed by main chain and side chain atoms of residues Phe140, Leu141, Asn142, Ser144, His163, His164, Met165, Glu166, His172 (Figs. 4d, 5). The aliphatic portion of the P1 glutamine side chain and terminating amide form remarkably conserved vdW, hydrophobic, and hydrogen bonding interactions along their length. In the product forms (comparison to acyl-enzyme intermediate forms will be treated separately below), Gln306 forms collectively seven largely conserved hydrogen bonds observed in all structures via side chain (Gln306 Oε1 to His163 Nε2, Gln306 Nε2 to Phe140 O, and Glu166 Oε1), mainchain (Gln306 NH to His164 O) and C-terminal carboxylate (Gln306 O to Gly43 NH and C145A NH of the oxyanion hole and Gln306 OXT to His41 Nε2) (Fig. 5; Supplementary Fig. 5). The strong complimentary for the S1 pocket may also promote desolvation effects. M^pro^ structures with an empty active site reveal consistently ordered waters that overlay conserved substrate hydrogen bond donor or acceptor groups (Fig. 7d). Binding of P1(Gln) would displace two waters typically observed in the S1 pocket and one in the oxyanion hole, adding further favorable entropic contribution to binding. We do note, however, in crystal structures determined at room temperature as opposed to more typical cryogenic temperature (100 K), these specific waters were less clearly resolved suggesting they are possibly more loosely bound^15,31^, an observation supported by minimal delta S of binding for small molecules to the S1 pocket^32^. Such observations provide valuable information to guide drug discovery as discussed below.

No major altered side chain amide rotamers are observed in our collective 52 structural visualizations of P1 over the varying substrate products or acyl enzyme intermediates, remarkably even amongst those with markedly varying main chain conformations such as C6 (form 2) (Fig. 5c and Supplementary Fig. 5). VdW interactions of the P1(Gln) with the S1 pocket residues are also remarkably conserved across the ensemble of cleavage site structures. A notable exception centers on the proximal side chain of Asn142 (Figs. 2, 3, 4d), with multiple rotamers observed. The planar amide side chain packs over the S1 pocket, facilitating multiple additional vdW interactions with the P1(Gln). Hydrogen bonds of the Asn142 amide nitrogen in the varying positions observed are at best long range. Given its potential to cap S1, encasing the P1 substrate side chain within, it is perhaps advantageous for the side chain of Asn142 to be untethered and dynamic to allow for post cleavage product release (see Fig. 4d). Our previous modeling of the ES complex^24^ further suggested a role for the P2′ substituent in influencing Asn142 positioning, enhancing the P1 stacked placement of the latter when S2′ is occupied due to steric repulsion, but allowing movement back towards that subsite upon product release, also enhancing product expulsion from the P1 site. Recent ES complexes using peptide substrates are in keeping with this, with similar Asn142 motions observed in response to occupancy of the S2′ prime site^20–23^. Collectively, it is not surprising therefore that the P1(Gln) is the most conserved feature of not only the family of coronaviral main protease cleavage sites but as well the substantial predicted human substrate repertoire recently identified through mass spectrometric based proteomics analysis^7,8^.

#### P2 position

The P2 position also plays a significant role in binding the substrate with a fascinating adaptation of the S2 pocket, specifically focused at the side chains of Met49 and Gln189, dependent on the nature of the P2 amino acid variant of the cleavage site (Figs. 2, 3, 4e, and 5). In our prior C5 product structure, with P2 phenylalanine, we observed the S2 subsite to form a relatively open conformation closely resembling the pocket makeup and dimensions compared to the majority of published apo structures^24^. We suggested this provides less energetically favorable binding post nsp5/nsp6 cleavage that likely plays a role in preventing self-inhibition of M^pro^ by its remaining C-terminal product. By far the most conserved residue at the P2 position though, in both viral and recently identified mammalian cleavage sites^7,8^, is leucine, present in all of our collective of structures with the exception of C5 (phenylalanine) and C6 (valine). Overlay of P2(Leu) shows remarked conservation of the aliphatic side chain and resultant vdW and hydrophobic interactions with the His41, Met49, Tyr54, His164, Met165, Asp187, and Gln189 side chain and main chain atoms forming the S2 pocket (Fig. 4e). P2 valine, unique to cleavage site C6 (Fig. 2d), also maintains a highly analogous set of interactions with a markedly similar S2 pocket, but with a less optimal vdW interaction surface as a result of the gap from the static pocket edge to the shorter beta-branched side chain of the P2(Val) C6 variant. This P2 pose is also remarkably maintained in the kinked form 2 structures, including C6 (form 2) (Fig. 2e) which we have captured in both binding orientations. This is despite the sometimes significant reorientations of the Met49 loop coincident with the close approach of the donor chain C-terminal domain.

The ensemble of structures here allows direct comparison of SARS-CoV-2 P2 substrate variants and provides additional atomic details specific to leucine induced rearrangement. Using C4 TSAV**L**Q as an example, the substrate adopts the same extended β-strand-like conformation as C5 SGVT**F**Q, with the P1-P4 backbone, in particular, superposing near perfectly and maintaining the common hydrogen bonds for P1 and P3 residues (Fig. 5a, b). P2(Leu) induces a distinct active site conformation in the S2 subsite with Met49 and Gln189 (and surrounding loops) changing orientation to create a deeper binding pocket in which the isobutyl side chain is buried, sandwiched between Met165 and Met49 (Figs. 2a, f, g, h, 3a–c, and 5a). Gln189 is more extended and forms a single hydrogen bond between side chain Oε1 and main chain NH of P2(Leu), an interaction common to all Leu/Val P2 substrates (Fig. 5a). In C5^24^, the Gln189 side chain amide instead forms two hydrogen bonds with the P4 main chain atoms (Figs. 2b,c, 5b).

#### P3 position

In the canonical form 1 binding pose (C4 for example, Fig. 2a, Fig. 5a), the P3 position of substrate points outwards towards the solvent, away from the binding groove. Despite this, the P3 position contributes two common β-strand hydrogen bonds with the main chain atoms of Glu166 that likely provides significant directionality, specificity and energetic contributions to substrate binding. The restriction to main chain interactions in P3 likely explains the low sequence conservation for this position, although it is perhaps surprising there is not as much variation in the P3 residues of the various substrates as one might expect for a surface residue (Fig. 1). Primarily small beta-branched residues, threonine or valine, are observed at P3, with C8, C9 and C10 having extended side chains with significant aliphatic character (lysine, arginine or methionine). The conservation of the smaller neutral or extended flexible side chains might indicate P3 has a conformational role in substrate binding or product release requiring a steric (side chain) preference, with moderate branched amino acid side chains such as leucine, aspartic acid, asparagine incompatible (and hinted at by our observations in the form 2 substrate interactions with the S4 pocket as below). Alternatively, or in addition, it may be the smaller neutral or extended flexible side chains are largely selected for to avoid steric clash with the conformationally labile neighboring residues along the binding groove that lie adjacent to the P3 side chain: specifically, Met49, Gln189, and Asn142 that mediate interactions with the anchoring P1 and sequence adapting (leucine/phenylalanine) for P2 as above.

#### P4 position

When the cleavage site region binds in a standard extended conformation (form 1 structures), the P4 residue occupies the complementary shallow and hydrophobic S4 binding pocket (side chains of Met165, Leu167, Pro168, and Gln192) which are remarkably closely positioned in all captured structures and in part influenced by the rigid Pro168 main chain conformation which packs directly against the Leu167 side chain. Consequently, the P4 position is dominated by the small residues alanine (4 occurrences) and valine (3), followed by threonine (2) and proline (2), which form hydrophobic contacts with Leu167 and Met165. P4 residues in all substrates except C5, which interacts with Gln189 Oε1 as described above, form a hydrogen bond between main chain NH and Thr190 O. In the non-classical (form 2) poses we observe here, the P3 side chains (Thr (C7, C13), Met (C10) or Arg (C14)) instead extend toward the S4 pocket due to rotation of the P3 main chain (Figs. 2e, i, 3d,e, and 5c), perhaps another reason for the restricted sequence diversity at P3. This observed plasticity in substrate binding orientation and adaptability between S3 and S4 subsites (in comparison to the closely fitting customized hydrophobic cave at P2 or conserved polar interactions of P1) could contribute to M^pro^’s ability to bind a diverse array of self and human substrates with considerable P4–P6 variability.

#### P5-6 positions

Finally, the highly sequence diverse P5 and P6 positions (Fig. 1) appear to play a minor role in direct binding and specificity with the enzyme active site. Unlike P1, P2 and P4, there appear to be no defined pockets for either of these terminal positions. P5 largely points away from the adjacent enzyme surface, specifically the enzyme loop 187–192 observed to be one of the 3 most highly mobile regions of the enzyme (Fig. 4a). In those form 1 substrate variants that we observe in the typical extended conformation, vdW interactions of the Cβ atoms of either the P5 residue with Thr190 and Ala191, and the equally diverse P6 residue with Pro168 and Ala191 side chains are the only observed of the limited interface at these positions, likely underlying their diversity. The exception is C5 (PDB 7JOY) where P6 Ser301 forms two long hydrogen bonds with mainchain N and O atoms of Gln192 via its O and Oγ atoms respectively (Fig. 5b and Supplementary Fig. 5). In the form 2 peptides, P5 and P6 are redirected between Met49 and Gln189, possibly due to the dramatic shift of the Gln189 side chain, reminiscent of motions observed in the C5 structure to accommodate the unique P2 phenylalanine (Fig. 5b, c). These structures highlight again the multiple roles and importance of this conserved amide side chain of M^pro^ in substrate specificity (Figs. 4e, 5c). Regarding the singular inability to capture C15 in our ensemble (also the least stable of our chimeric constructs), the unique presence of bulky aromatic residues at both P6 (Tyr) and P5 (Phe) suggests possible steric interference prohibiting even the minimal contacts observed for the other cleavage sites. This in combination with the P4 (Pro) and P3(Lys) may suggest added conformational flexibility/non-optimized S4 binding (compared to canonical and as with the only other P4(Pro) containing C10 variant) and perhaps weakened binding for this site that underlies the inability to capture it in crystallo. Future systematic analysis of a unified set of M^pro^ cleavage site kinetic data will provide the needed further support for these fascinating sequence-specific variations in binding.

### Acyl-enzyme and product structures of C12 PHTVLQ

**Fig. 6:**
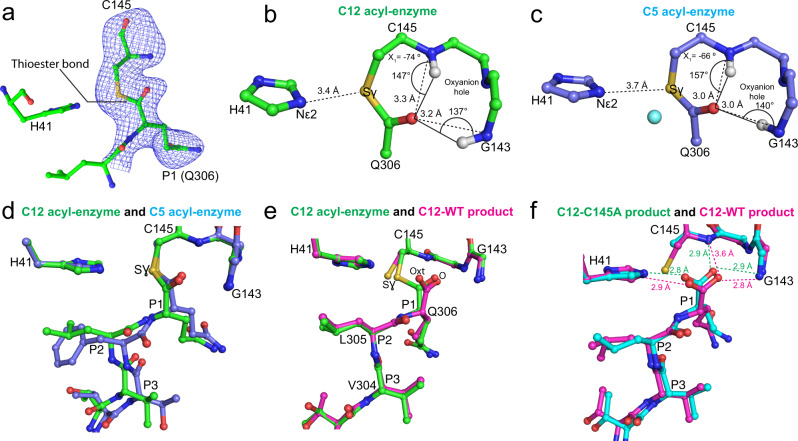
Characterization of wild-type M^pro^ C12 acyl-enzyme complex structure and comparison to wild-type C12 product complex and C5 acyl-enzyme complex. **a** mF_o_-DF_c_ omit electron density map (contoured at 3.0 σ, blue mesh) shows the thioester bond between the mainchain carbonyl carbon of Gln306 (chain C; residues Leu305 and Gln306 shown) and the Sγ of Cys145 within the wild type M^pro^ catalytic site (chain B; His41 and Cys145 shown) of C12. The ball-and-stick structure is shown with carbon green, nitrogen blue, sulfur gold, and oxygen red. **b** Analysis of the C12 acyl-enzyme structure (chains B and C). Ball-and-stick (carbon green, nitrogen blue, oxygen red, sulfur gold) view shows the geometry and atomic interactions of the thioester bond between the Sγ of Cys145 and main chain carbonyl carbon of Gln306. The trigonal planar thioester group, defined by atoms Cα, C, and O of Gln306, and Sγ of Cys145 is shown as is the χ_1_ dihedral angle (defined by atoms N, Cα, Cβ, and Sγ). The oxyanion hole hydrogen bond distances and angles are shown. **c** Analogous analysis of the acyl-enzyme intermediate of C5 (chain B and symmetry-related chain B) with ball-and-stick view shown (carbon light blue, nitrogen blue, oxygen red, sulfur gold). The proposed deacylating water is shown as a cyan sphere. **d** Superposition of the C12 WT acyl-enzyme complex (green carbons, chains B and C) and C5 WT acyl-enzyme complex (light blue carbons, chain B, and symmetry-related chain B) complexes. **e** Superposition of the C12 WT acyl-enzyme complex (green carbons, chains B and C) and the C12 WT product complex (magenta carbons, chains D and E). **f** Superposition of the C12 C145A product complex (cyan carbons, chains B and C) and the C12 WT product complex (magenta carbons, chains D and E).

Unlike the C145A mutant, obtaining crystals with the C-terminal P6-P1 substituted sequences bound in the wild-type catalytic Cys145 active site was more difficult, with only two examples observed for the C12 and C13 variants. In the C12 PHTVLQ WT structure (Supplementary Table 3), the ~2.3 Å resolution map showed the clear presence of a covalent acyl-enzyme intermediate (Fig. 6a and Supplementary Fig. 1). Given the nature of the C-terminal product-like complexes here, observation of an intermolecular covalent link between catalytic Cys145 and Gln306 from a different M^pro^ homodimer demonstrates that this reaction has occurred in reverse from product to acyl enzyme states. We suggest this is likely afforded by the protein-tethered substrate and resulting high effective local concentration, a condition much harder to capture with the typical truncated peptide substrates used in the majority of protease studies. Remarkably, a non-covalently bound C-terminal product was also observed in the same crystal, but in a distinct active site in a second functional dimer of the 12 molecules of the ASU, providing the direct comparison of a product and acyl enzyme substrate complex both in the native active site context and identical crystallization conditions. An additional structure of C12 in complex with the C145A mutant in the same isomorphous crystal form was also characterized here. These structures collectively allow both comparison with our previously determined acyl-enzyme intermediate of C5^24^ and, importantly, subtle but significant differences with how the cleaved product would bind the native active site in presence of Cys145 (as opposed to alanine in the C145A or H41A mutant forms used for all previous capture of product complexes in either SARS-CoV-1 or -2).

Native and C145A C12 variant crystals belong to space group P2_1_ with six dimers in the asymmetric unit. In both, the C-terminal C12 PHTVLQ substrate sequence is clearly resolved in the equivalent active sites of four protomers. In the WT structure, careful analysis of the electron density for the native structure supports both the covalent acyl-enzyme and non-covalent product carboxylate binding of Gln306 in distinct active sites (Fig. 6a and Supplementary Fig. 1). For the acyl-enzyme intermediate, the thioester bond formed between the Cys145 γ-sulfur atom and the carbonyl carbon of P1(Gln) creates a trigonal planar linkage group (defined by atoms Cα, C, and O of Gln306, and Sγ of Cys145), with Cys145 χ_1_ angles (defined by atoms N, Cα, Cβ, and Sγ) of ~ −70/−74^ο^ (Fig. 6b, c), consistent with our previous observation for the C5 acyl-enzyme intermediate complex^24^ (−66^ο^). The conserved oxyanion hole hydrogen bond distances and angles, a central stabilizing force in substrate carbonyl polarization, and subsequent oxyanion transition state stabilization, are also shown. In neither of the two acyl enzyme complexes is there evidence in the electron density for the catalytic water as captured in the earlier C5 acyl enzyme complex^24^. We also see a significant differential in distance of the His41 imidazole nitrogen Nε2 to the Cys145 Sγ it activates during acylation. In our C12 structures here, this distance is 3.4 Å in both acyl enzymes, whereas in the prior C5 structure it is longer at 3.7 Å. We propose that the pH of crystallization in these two variants (pH 7 for C12, pH 6 for C5) has affected the protonation state of the catalytic general base His41; in the C5 structure, a protonated state of the imidazole would be favored at the lower pH, allowing the deacylating water to be left unactivated and captured in that prior structure. In the C12 variants here, the higher pH would favor deprotonation of the His41 Nε2, allowing closer approach to the Cys145 Sγ (Fig. 6b–d) and we propose consequent steric occlusion of the catalytic water, collectively indicating how pH and catalytic group pK_a_s need to be considered to capture these complexes.

As mentioned, the electron density in the other two chains in the C12 WT crystal with bound C-terminus supports the predominant presence of the product carboxylate instead of the acyl-enzyme. An overlap of the two mechanistic snap shots captured under identical crystallization conditions (Fig. 6e), highlights the consequential rotation in the product complex of the Sγ atom (χ_1_ angle of ~ −90^ο^), to avoid steric and electrostatic repulsion with the negatively charged carboxylate oxygens of the product. The concomitant apparent destabilization of the product carboxylate by loss of critical electrostatic interactions with the positive partial charges of the oxyanion hole main chain nitrogens is also observed, with notably the loss of a hydrogen bond to the Cys145 backbone amide (3.6 Å from Gln306 O to Cys145 NH, Fig. 6f). We propose these observed motions and destabilization in the WT context, are necessary to effect substrate product release which must occur efficiently post cleavage. These observations contrast with the binding of C12 to the C145A mutant active site, where the lack of the Cys145 γ-sulfur atom allows a more optimum interaction network between P1(Gln) with the oxyanion hole, S1 subsite, and His41 (Fig. 6f). This observed more stable interaction of P1(Gln) with the C145A mutant provides an explanation why it has been much easier obtaining these complexes compared to the native active site.

Finally, an additional product complex has been observed in the C13 WT crystal. Interestingly, in this case, the electron density unambiguously shows the Cys145 γ-sulfur atom to be oxidized (Supplementary Fig. 1), resulting in an even more dramatic steric/electrostatic repulsion of the product carboxylate out of the complimentary oxyanion hole (both oxyanion hole hydrogen bonds lost as well as the stabilizing interactions with His41 Nε2 and vdW contacts with side chain of Asn142). Remarkably, despite this significant displacement from optimal binding, the P1(Gln) side chain remains bound in S1 with its side chain vdW and hydrogen bonding interactions maintained (Fig. 3d), as well as the P2(Leu), illustrating again the anchoring power of these positions and importance in M^pro^ substrate cleavage.

### Implications for drug discovery

SARS-CoV-2 M^pro^ is a major focus for the development of direct-acting antivirals (DAA) to treat COVID-19. A detailed understanding of native substrate recognition as reported here provides valuable information on targeting the active site structure and mechanistic features.

Early in the pandemic, a concerted drug-repurposing effort identified several promising covalent acting peptidomimetics, which reproduced the binding of the native substrates with a covalent warhead hijacking the active site cysteine nucleophile. These included DAAs previously developed for SARS-CoV-1^9,10,33,34^, other coronaviruses^35,36^, or other viral proteases^36,37^. In particular, Pfizer has developed two inhibitors that are amongst the most promising current DAAs targeting M^pro^. PF-07304814, and its active metabolite PF-00835231 (Supplementary Fig. 6), is an IV-administered ketone-based covalent cysteine protease inhibitor that was initially developed against SARS-CoV-1^34^ and was found to maintain potent inhibition against SARS-CoV-2^33^. Nirmatrelvir (PF-07321332) (Supplementary Fig. 6) was subsequently developed and is a reversible covalent inhibitor that utilizes a nitrile warhead to target the catalytic cysteine^18^. It has the advantage of being orally administered and demonstrated a ~90% reduction in risk of hospitalization in patients with mild to moderate COVID-19 in initial clinical trials^5^. Co-administered with HIV antiretroviral ritonavir under the name Paxlovid, it has been granted both FAA and EMA authorization and represents the first approved DAA for COVID-19 targeting M^pro^.

**Fig. 7:**
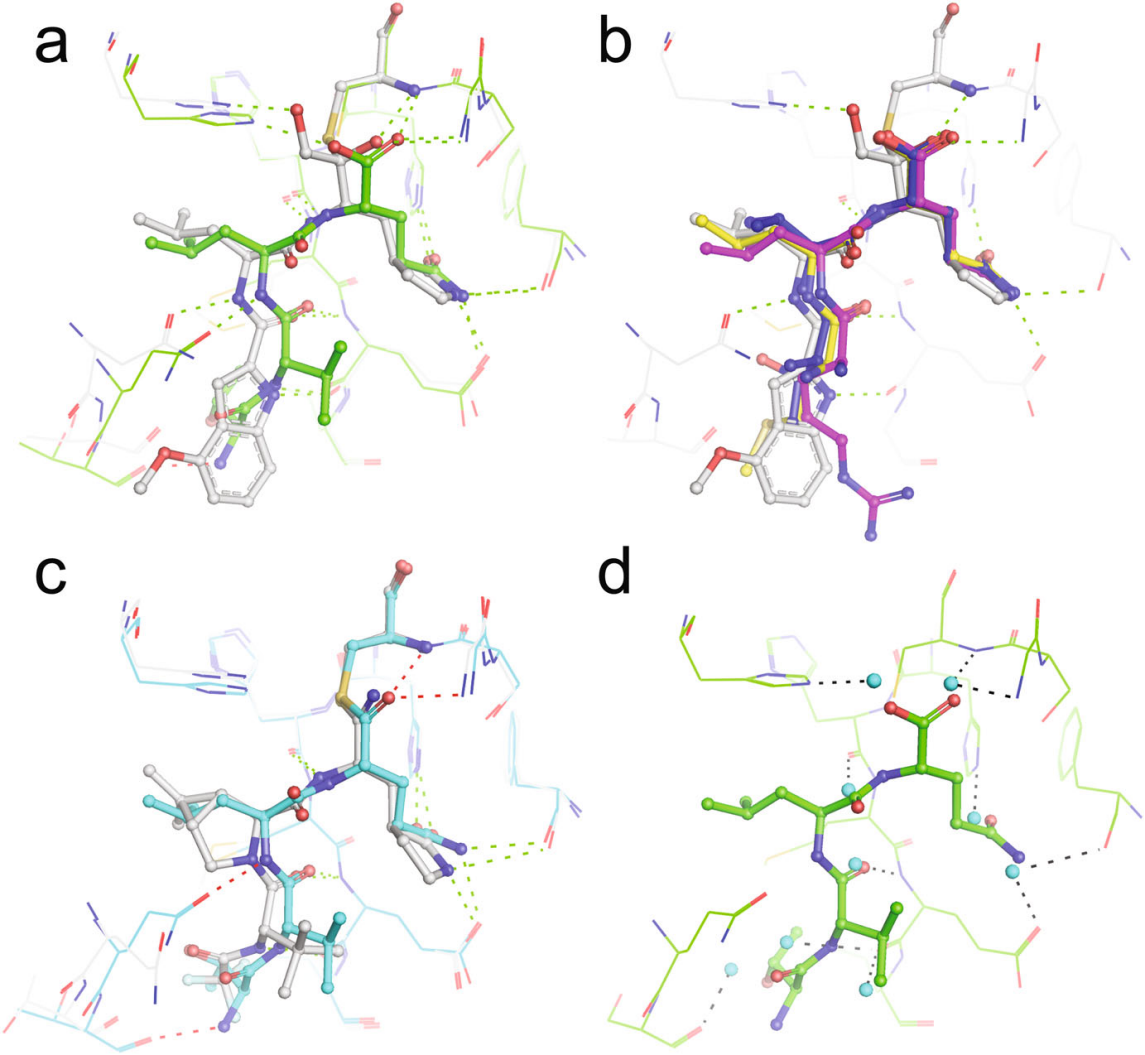
Clinical direct-acting antivirals (DAAs) mimic substrate binding to the SARS-CoV-2 M^pro^ active site. **a** PF-00835231 (gray; PDB 6XHM^33^) superposed with C12 product (green). Hydrogen bonds common to inhibitor and product colored green, hydrogen bonds unique to product red. **b** PF-00835231 (gray) superposed with C6 (form2) (blue), C10 (yellow) and C14 (magenta) products, which adopt the kinked form 2 conformation. The product P3 residue projects across the S3 and S4 pockets similar to the capping indole in PF-00835231. Hydrogen bonds common to inhibitor and product colored green. **c** Nirmatrelvir (gray; PDB 7RFS^18^) superposed with C12 acyl enzyme intermediate (cyan). Hydrogen bonds common to inhibitor and product colored green, hydrogen bonds unique to product red. **d** Commonly observed waters (cyan spheres) in the empty M^pro^ active site overlaid on the C12 product structure, showing their apparent displacement upon substrate binding. Hydrogen bonds common with product are shown in black. The M^pro^ water positions were assessed using PDB 6YB7^17^ and 7JOY^24^.

Comparison of PF-00835231 and nirmatrelvir binding to the common substrate interactions identified here explains their potency and rationale for the drug-development between the two compounds. Both mimic binding of substrates with P2(Leu) (C12 P4-P1 TVLQ overlaid in Fig. 7a, c for comparison with acyl enzyme and product structures). The P1 2-pyrrolidinone (a common P1(Gln) surrogate found in the majority of peptidomimetic compounds) is bound in the S1 pocket and reproduces the key conserved interactions we observe for all substrates (Fig. 7a). In PF-00835231, a leucine binds the S2 pocket and forms the common hydrogen bond between Gln189 Oε1 and the P2/P3 amide bond observed in P2(Leu) structures here (Fig. 7a). The capping indole nitrogen and preceding carbonyl oxygen form the conserved hydrogen bonds with the backbone atoms of position Glu166, and extends toward, but does not engage, the S4 pocket like the canonically posed native substrates. To improve the poor bioavailability of PF-00835231, initial designs aimed to reduce the number of hydrogen bond donors^18^. A nitrile warhead replaced the α-hydroxymethyl ketone moiety, losing the hydrogen bond with His41 Nε2 that is consistently observed here for the product carboxylate. The thioimidate intermediate adopts trigonal planar geometry resembling the acyl-enzyme intermediate structures of C5 and C12; however, the imino NH is unable to form the typical oxyanion backbone hydrogen bond interactions seen with the native acyl enzyme (Fig. 7c). Borrowing directly from the HCV antiviral boceprevir, which also exhibits potent inhibition of SARS-CoV-2 M^pro^^36,38,39^, and earlier drug development efforts^40–42^, a 6,6-dimethyl-3-azabicyclo[3.1.0]hexane was introduced as a leucine surrogate at P2 and the indole group was replaced with a branched, acyclic group at P3, similar to the consensus small aliphatic/polar residues. The loss of the hydrogen bond between the P2/P3 amide linkage (now cyclized) and Gln189, an interaction common to all Leu P2 structures observed here, resulted in a marked loss of potency but with desired increased oral absorption. Finally, a trifluoroacetamide was introduced at P4 with effective engagement of the S4 binding pocket (Fig. 7c), with a corresponding improvement in inhibitor potency.

The observed recovery of potency with improved mimicry of substrate P3 and P4 positions prompted us to explore this further. We analyzed the contribution of P3 interactions in the Postera database^43^ looking at the two conserved main chain hydrogen bonds with the Glu166 backbone. Although no compounds reproduced both interactions simultaneously, potent noncovalent compounds form a hydrogen bond with the Glu166 backbone nitrogen. We note this critical hydrogen bond is also conserved in all of our kinked (form 2) substrate structures as well as canonical poses, highlighting its importance in substrate binding (Fig. 7c). Demonstrating the contribution of the P4 group to the overall binding affinity of noncovalent inhibitors, Zhang et al.^44^ and Deshmukh et al.^45^ reported development of a series of perampanel derivatives where the P1, P2 and P1′ groups were maintained and the majority of analog design focused on the P4 group. Addition of secondary extended groups that productively engage the S4 pocket resulted in significant increase in inhibitor potency from 5 to 10 μM to low nM. Strong hydrophobic interactions induced by the presence of a halogen in the S4 corroborate our observations of requiring a small hydrophobic residue at P4 in the native substrates.

Collectively, our structures and analyses reveal the key ensemble of interactions common to substrate binding, the role of which have been substantiated by existing DAA development. In particular is the importance of considering not only the critical anchoring interactions formed with the S1 and S2 pockets, but also downstream P3 main chain and S4 subsites which contribute significant free energy to substrate binding. These common and conserved interactions will also be important when considering the potential for the emergence of drug resistance, a hallmark of HIV antiviral therapy which similarly focussed on a viral protease. Although no M^pro^ mutations in current SARS-CoV-2 variants have been described that impact existing DAAs^46^, the accumulation of mutations at ~95% of positions throughout the M^pro^ sequence^47^ highlight a potential future problem. Identification of the key substrate binding interactions that would be less prone to variation will be an important consideration to minimize the likelihood of resistance arising. These include both maximizing interactions at sites that accumulate fewer mutations, so called evolutionary cold-spots^47^. In the M^pro^ active site this includes for example His163 in the S1 pocket, which forms a common hydrogen bond with Gln P1 in all substrates, and hydrophobic residues that make up the S4 pocket (Leu167, Phe185, and to a lesser extent Met165)^47^. Interestingly, residues Asn142, Met49 and the loop containing Gln189 (188–191), regions here shown to be highly mobile, are positions that tend to accumulate significant number of mutations and consequently likely potential areas for resistance to develop^47^. In addition, exploiting the conserved backbone interactions along the length of the active site, where the interaction is not contingent on the specific amino acid side chain, would reduce the chance of mutations leading to resistance. In our structure ensemble, these are shown to be particularly important for binding with four common backbone interactions observed for the P1, P3, and P4 substrate positions (Fig. 5, Supplementary Fig. 5, and Supplementary Table 7).

We believe the collective of high occupancy substrate structures captured here provide a valuable resource for further understanding of M^pro^ essential action in viral maturation, ability to cleave and presumably modulate multiple human targets during disease progression, and further inform the structure-guided design of drugs to tackle the global specter of COVID-19.

## Methods

### Cloning, protein production, and purification of M^pro^ cleavage site variants

The gene-encoding full-length SARS-CoV-2 M^pro^ (UniProt P0DTD1) was cloned into a modified pET-28a plasmid including an N-terminal dual His-SUMO tag (Supplementary Table 1). Mutant C145A was generated using QuickChange site-directed mutagenesis. A series of C-terminal mutant chimeras were individually generated in context of both WT and C145A mutant backgrounds, replacing the M^pro^ C-terminal 6 residues (residues 301–306 corresponding to the nsp5/nsp6 P6–P1 cleavage sequence C5) with the equivalent P6-P1 sequence from the 10 other M^pro^ cleavage sites within polyprotein pp1ab. The resulting mutant constructs were confirmed by DNA sequencing (see Supplementary Table 1 for the SUMO M^pro^ WT sequences and a list of primers used for mutagenesis).

Protein expression was carried out in *E. coli* BL21 (DE3). Cells were grown at 37 °C in LB media supplemented with 0.05 mg/mL kanamycin. At an OD600 of ~1, protein expression was induced with the addition of IPTG to a final concentration of 1 mM. Cells were harvested after 3 h, resuspended in lysis buffer (50 mM Tris pH 7.4, 300 mM NaCl), and lysed with an Avestin Emulsiflex C5. The lysate was centrifuged at 50,000 × *g* for 45 min, and the soluble protein was loaded onto a gravity flow column packed with 5 mL HisPur Ni-NTA resin (ThermoFisher Scientific) equilibrated in the lysis buffer with 20 mM imidazole. The column was washed with 5 column volumes of lysis buffer and 5 column volumes of the buffer with 40 mM imidazole, and then eluted with 50 mM Tris pH 7.4, 300 mM NaCl, and 300 mM imidazole. The eluate was dialyzed overnight at 4 °C in 1.5 L 50 mM Tris pH 7.4, 300 mM NaCl with ~1 mg/ml SUMO protease to leave the native N-terminal M^pro^ sequence. Uncleaved His-SUMO-M^pro^, cleaved His-SUMO and His-tagged SUMO protease were removed with 0.5 mL HisPur Ni-NTA resin before further purification by gel filtration chromatography with a Sephacryl S-200 HR 16/60 column (GE Healthcare) equilibrated in 50 mM Tris pH 7.4, 1 mM EDTA, and 1 mM DTT. This protein was concentrated by ultrafiltration (Amicon Ultra-30; Millipore Sigma) to >10 mg/mL and frozen in liquid nitrogen for storage at −80 °C. Final concentration was determined by absorbance at 280 nm using the extinction coefficient of 32890 M^−1^ cm^−1^, see Supplementary Table 2.

### Crystallization of M^pro^ cleavage site variants

Crystallization trials were undertaken using a Mosquito LV (SPT Labtech) crystallization robotics system with commercially available crystallization screens (Classics, JCSG+, PACT; Qiagen). Screens were carried out in sitting drop INTELLI-PLATE 96 well plates (Art Robbins Instruments) with drops composed initially of 0.5 µl of protein in conditions as above mixed with 0.5 µl mother liquor reservoir solution. Where necessary, crystal hits were further optimized by varying conditions surrounding the pH, salt, or precipitant. Final crystallization conditions for each cleavage site complex are provided in Supplementary Table 2. For all crystals, cryoprotection during data collection was implemented by raising, as needed, the precipitating agent concentration in the drop (various PEGS) to 35% prior to flash freezing in liquid nitrogen. In other conditions, 30% glycerol was used as a cryoprotectant.

### X-ray crystallographic structure determination of M^pro^ cleavage site variants

Diffraction data were collected at 100 K on beamlines CMCF-BM at the Canadian Light Source using MxDC for data collection, 23-ID-B and 23-ID-D at the Advanced Photon Source using JBlueIce for data collection, or beamlines 5.0.1 and 5.0.2 at the Advanced Light Source using b4 for data collection (see Supplementary Table 2). Diffraction data were processed using xia2^48^ and XDS^49^, with data reduction carried out using Aimless^50^ as part of the CCP4 package^51^ (see Supplementary Table 3), data were corrected for anisotropic diffraction using the STARANISO server (http://staraniso.globalphasing.org/cgi-bin/staraniso.cgi; Supplementary Tables 4a and 4b). Phasing was carried out using molecular replacement with PDB 7JOY, chain B as the search model in Phaser^52^, also part of the CCP4 package. Sequential rounds of model building and refinement were carried out using Coot^53^ and Phenix refine^54^. Models from the same space group were placed on a standard origin using the ACHESYM server^55^. Validation of the final models was carried out using MolProbity^56^ with excellent model stereochemical statistics; see Supplementary Table 3.

All structure analysis and figure preparation were carried out with PyMOL (The PyMOL Molecular Graphics System, Version 2.1, Schrödinger, LLC) and Coot, distributed as part of the CCP4 package. M^pro^-substrate interfacial hydrogen bonds and surfaces (default probe radius of 1.4 Å) were also analyzed by PISA^27^ and cross checked with CONTACT in the CCP4 package^51^. Electron density maps for figures were generated using Phenix^54^ with OMIT maps calculated with phenix.polder^57^. Structural alignments were performed using the ALIGN function in PyMOL with all protein atoms.

### Reporting summary

Further information on research design is available in the Nature Research Reporting Summary linked to this article.

## Supplementary information

Supplementary Information

Reporting Summary

## Data Availability

The data that support this study are available from the corresponding authors upon request. Structure factors and atomic coordinates have been deposited with the protein data bank with accession codes 8DRR, 8DRS, 8DRT, 8DRU, 8DRV, 8DRW, 8DRX, 8DRY, 8DRZ, 8DS0, 8DS1, 8DS2.
