# Supplementary material for: X-ray crystallographic characterization of the SARS-CoV-2 main protease polyprotein cleavage sites essential for viral processing and maturation
Source: Nat Commun. 2022 Sep 3;13:5196. doi: 10.1038/s41467-022-32854-4 (PMC9440467; doi:10.1038/s41467-022-32854-4)
Supplement: Supplementary file 1 — Supplementary Information [file 41467_2022_32854_MOESM1_ESM.pdf]

Supplementary Information

X-ray crystallographic characterization of the SARS-CoV-2 main protease polyprotein cleavage sites essential for viral processing and maturation

Jaeyong Lee[1,2*], Calem Kenward[1*], Liam J. Worrall[1*], Marija Vuckovic[1], Francesco Gentile[3], Anh-Tien Ton[3], Myles Ng[1], Artem Cherkasov[3], Natalie C.J. Strynadka[1†] and Mark Paetzel[2†]

[1]Department of Biochemistry and Molecular Biology and Centre for Blood Research, The University of British Columbia, Vancouver, British Columbia, Canada; [2]Department of Molecular Biology and Biochemistry, Simon Fraser University, Burnaby, British Columbia, Canada; [3]Vancouver Prostate Centre, The University of British Columbia, Vancouver, British Columbia, Canada

*These authors contributed equally.

† Correspondence to ncjs@mail.ubc.ca and mpaetzel@sfu.ca

This file includes:

Supplementary Figures 1-6 and Supplementary Tables 1-7

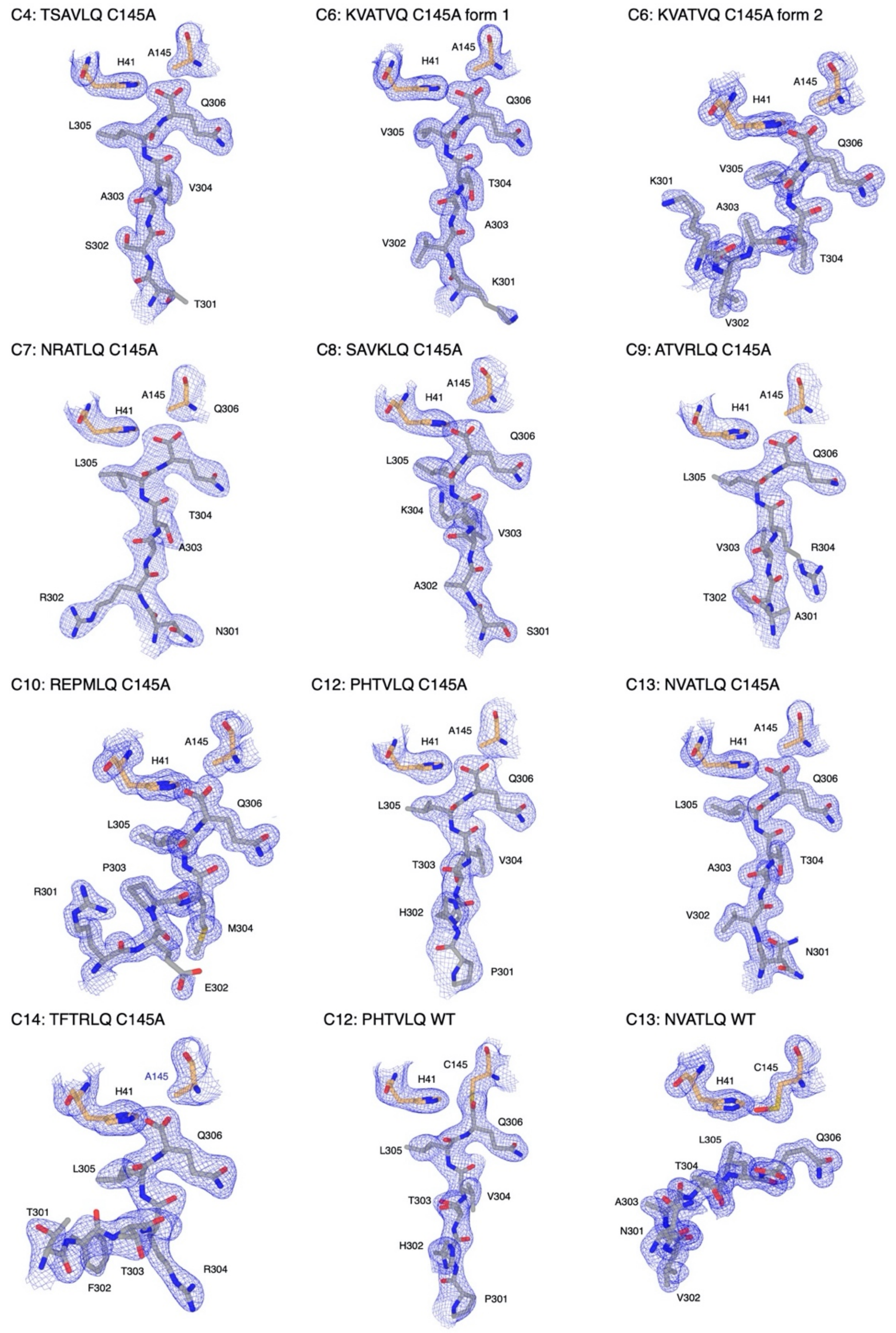

Supplementary Fig. 1. Representative electron density maps for bound cleavage sites. 2mFo-DFc electron density (contoured at 1.0 σ) of each substrate sequence (C4-C14) shows presence of the bound C-terminal product within catalytic site of wildtype (C12-WT and C13-WT) and C145A (C4-C14) M^{pro} mutants.

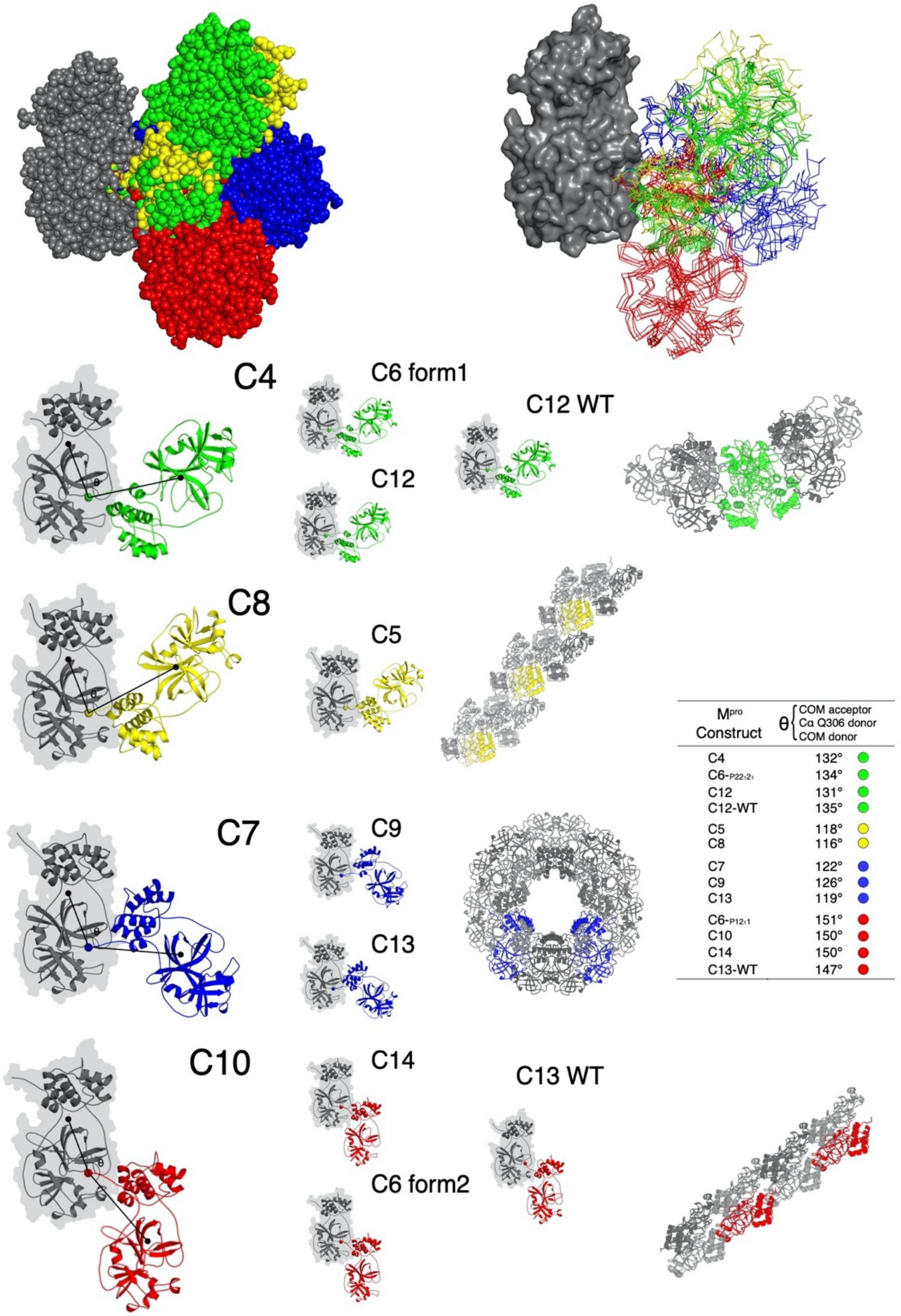

M^{pro} Construct	θ { COM acceptor / Cα Q306 donor / COM donor	
C4	132°	● (green)
C6-$_{P22_12_1}$	134°	● (green)
C12	131°	● (green)
C12-WT	135°	● (green)
C5	118°	● (yellow)
C8	116°	● (yellow)
C7	122°	● (blue)
C9	126°	● (blue)
C13	119°	● (blue)
C6-$_{P12_11}$	151°	● (red)
C10	150°	● (red)
C14	150°	● (red)
C13-WT	147°	● (red)

Supplementary Fig. 2. The angles of approach that the C-terminus of donor protomers make towards the substrate specificity binding groove of its neighboring acceptor molecule. Donor protomers are colored and neighboring acceptor molecules are grey with a common orientation. Four different general approach clusters were observed for the presentation of the specificity residues (colored green, blue, yellow, and red) to the neighboring active site (colored grey) and as superposed collectively in the space filling surface diagram, top left, with corresponding superposition of donor line drawings right. The four approach clusters are further depicted individually. The Cα of residue 306 in each grouping is shown as a sphere to highlight its position within the active site of the statically placed acceptor. The corresponding crystal lattice packing is also illustrated adjacent for each grouping. The calculated angle of approach is defined in the inset table (COM: Center of Mass).

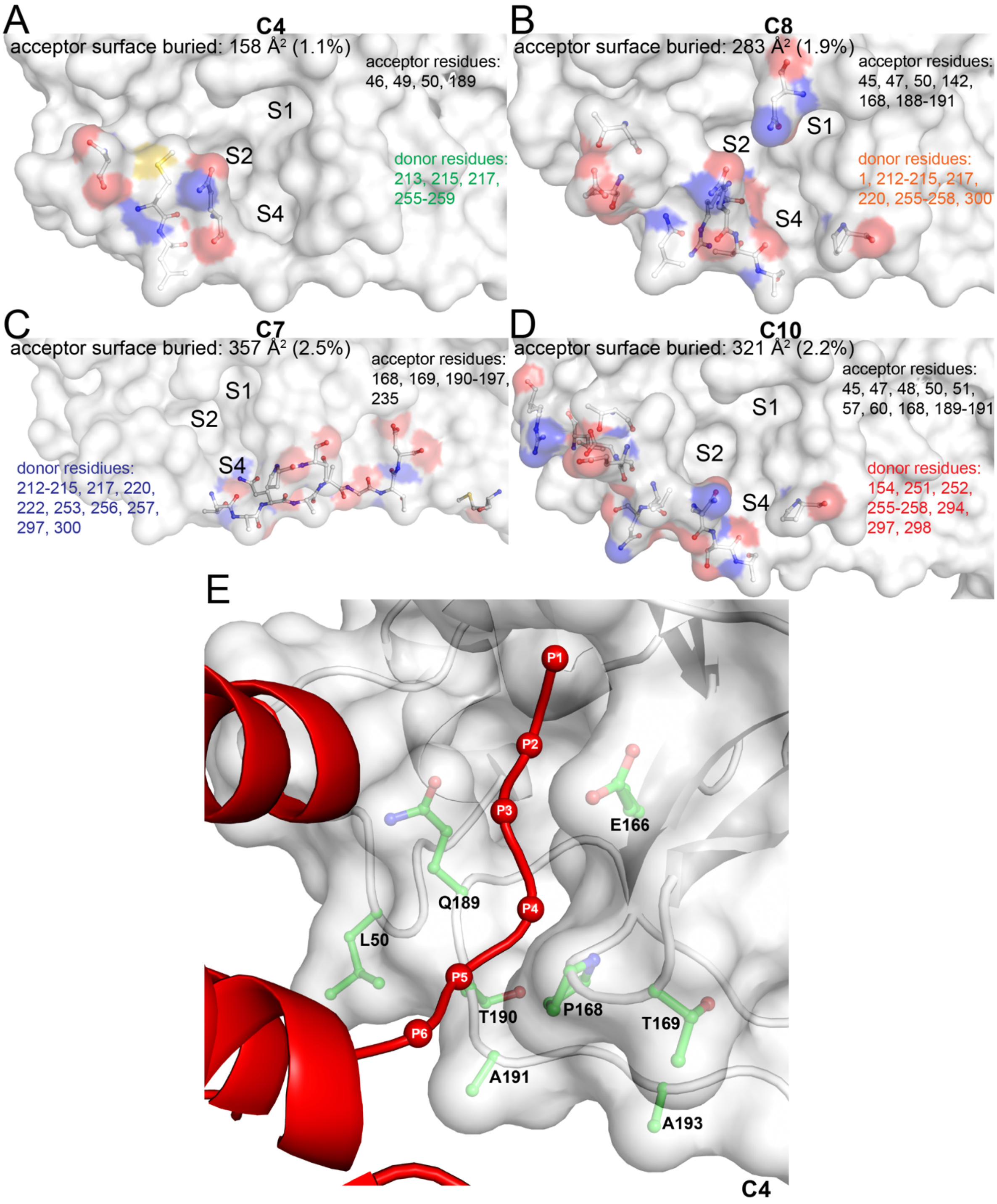

Supplementary Fig. 3. Approach variant binding analysis. A-D Contact surface illustration and analysis of the M^pro^ donor/acceptor approach variants as shown and colored in Supplementary Fig. 2 highlighting their varied positioning on either side of the acceptor binding groove cleft (labelled subsites). The acceptor and donor residues involved (P6-P1 excluded), the buried surface as well as number of productive hydrogen bonds are listed in each case. **E.** A zoomed in view of the conserved aliphatic ridge at the entrance of the M^pro^ acceptor binding groove we hypothesize may ensure an open form for facilitated binding of multiple viral and human substrates. C4 donor is shown in red ribbon with C-terminal P6-P1 Cα atoms denoted as red spheres, localized in the acceptor active site groove (grey molecular surface superposed with grey Cα ribbon, highlighted side chains in CPK with green carbons and labelled).

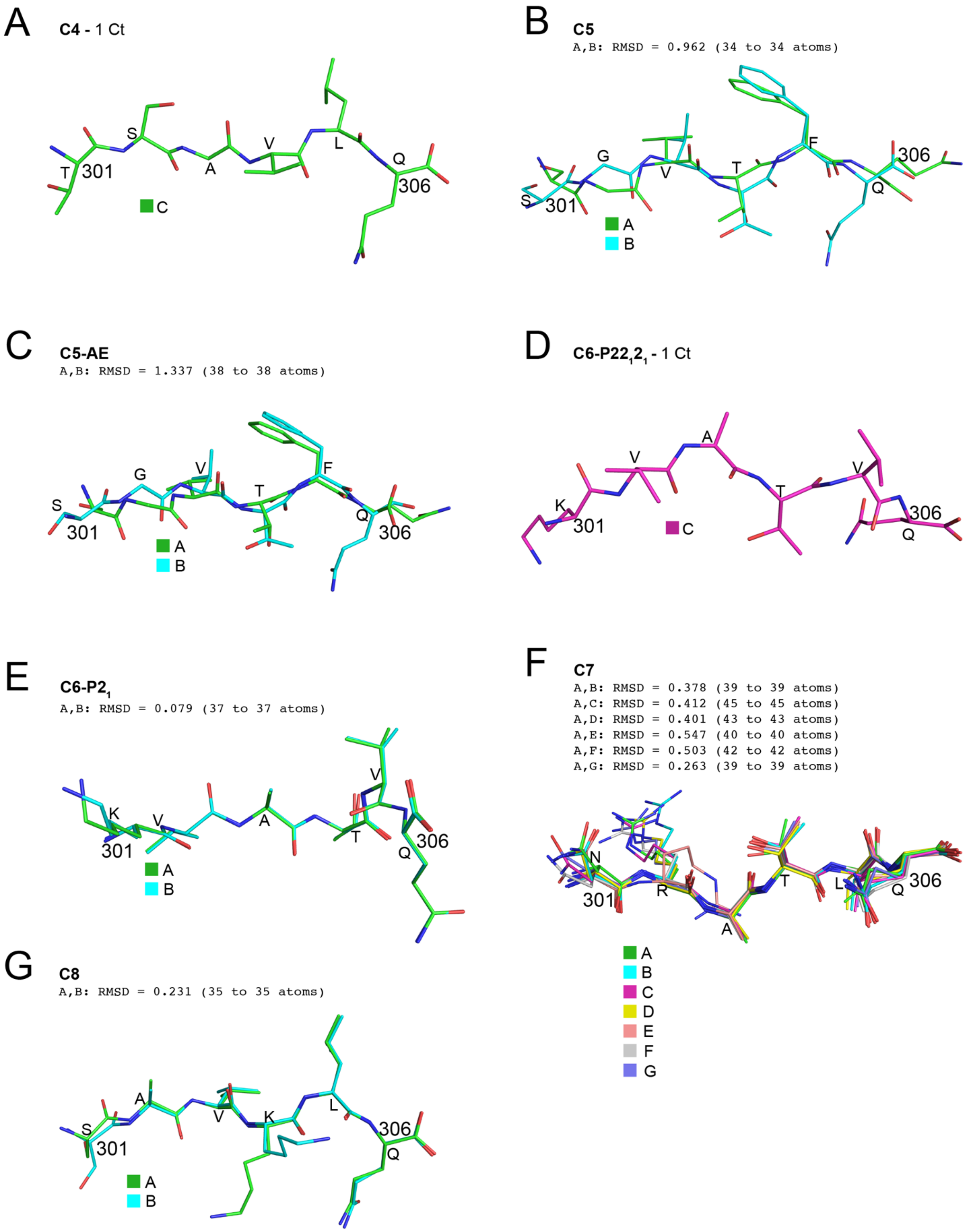
A
C4 - 1 Ct
T 301 S A V L Q 306
C
B
C5
A,B: RMSD = 0.962 (34 to 34 atoms)
S 301 G V T F Q 306
A
B
C
C5-AE
A,B: RMSD = 1.337 (38 to 38 atoms)
S 301 G V T F Q 306
A
B
D
C6-P22$_1$2$_1$ - 1 Ct
K 301 V A T V 306 Q
C
E
C6-P2$_1$
A,B: RMSD = 0.079 (37 to 37 atoms)
K 301 V A T V Q 306
A
B
F
C7
A,B: RMSD = 0.378 (39 to 39 atoms)
A,C: RMSD = 0.412 (45 to 45 atoms)
A,D: RMSD = 0.401 (43 to 43 atoms)
A,E: RMSD = 0.547 (40 to 40 atoms)
A,F: RMSD = 0.503 (42 to 42 atoms)
A,G: RMSD = 0.263 (39 to 39 atoms)
N 301 R A T L Q 306
A
B
C
D
E
F
G
G
C8
A,B: RMSD = 0.231 (35 to 35 atoms)
S 301 A V K L 306 Q
A
B

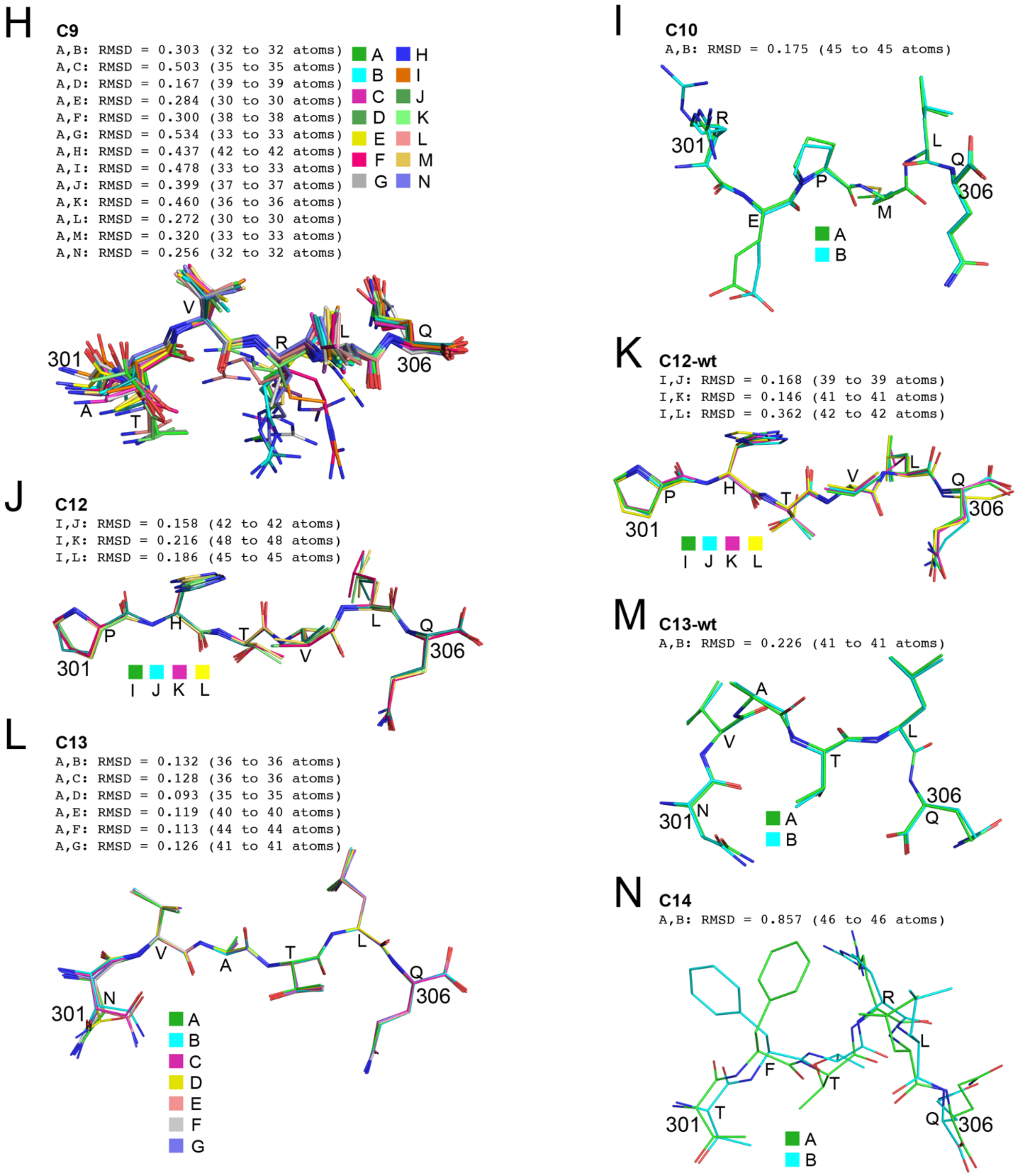

Supplementary Fig. 4. Structural superposition of residues P6 (301) through P1 (306) for all protomers within the asymmetric unit of each cleavage site variant crystal. Listed above the line structure of the superimposed chains are the root mean square deviation values for each chain and the number of common atoms applied to the alignment onto chain A. Each chain has a different color for the carbons which is defined by the legend below each alignment. Each residue is labeled. All resulting RMSD values are listed in Å units.

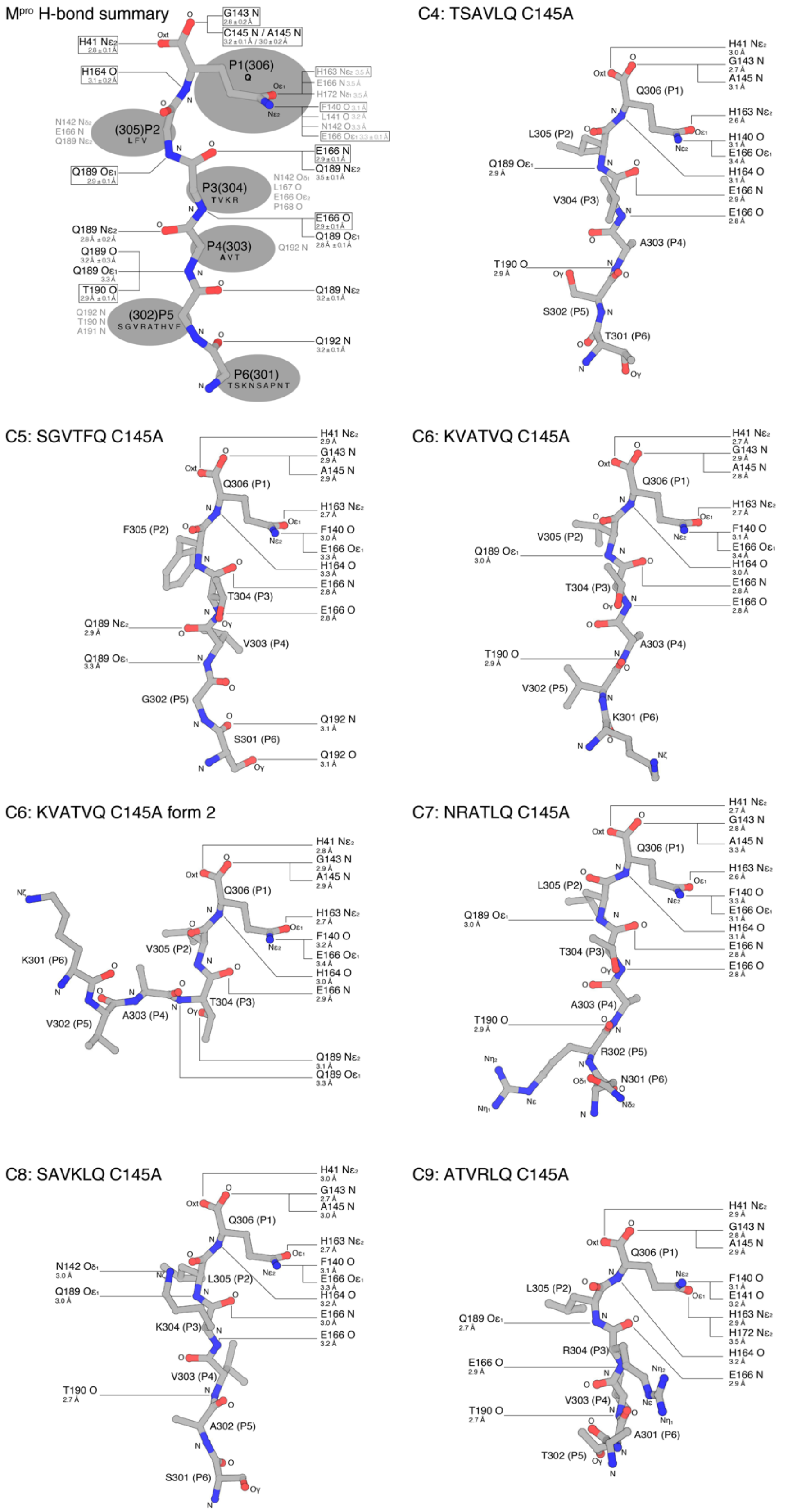
Mpro H-bond summary
G143 N
C145 N / A145 N
H41 Nε2
H164 O
P1(306)
Q
H163 Nε2
E166 N
H172 Nδ1
F140 O
L141 O
N142 O
E166 Oε1
(305)P2
LFV
N142 Nδ2
E166 N
Q189 Nε2
E166 N
Q189 Nε2
Q189 Oε1
P3(304)
TVKR
N142 Oδ1
L167 O
E166 Oε2
P168 O
E166 O
Q189 Oε1
Q189 Nε2
P4(303)
AVT
Q192 N
Q189 O
Q189 Oε1
T190 O
Q189 Nε2
Q192 N
T190 N
A191 N
(302)P5
SGVRATHVF
Q192 N
P6(301)
TSKNSAPNT
C4: TSAVLQ C145A
H41 Nε2
G143 N
A145 N
Q306 (P1)
H163 Nε2
L305 (P2)
H140 O
E166 Oε1
Q189 Oε1
H164 O
V304 (P3)
E166 N
E166 O
A303 (P4)
T190 O
S302 (P5)
T301 (P6)
C5: SGVTFQ C145A
H41 Nε2
G143 N
A145 N
Q306 (P1)
H163 Nε2
F305 (P2)
F140 O
E166 Oε1
H164 O
E166 N
T304 (P3)
E166 O
Q189 Nε2
V303 (P4)
Q189 Oε1
G302 (P5)
Q192 N
S301 (P6)
Q192 O
C6: KVATVQ C145A
H41 Nε2
G143 N
A145 N
Q306 (P1)
H163 Nε2
V305 (P2)
F140 O
E166 Oε1
Q189 Oε1
H164 O
T304 (P3)
E166 N
E166 O
A303 (P4)
T190 O
V302 (P5)
K301 (P6)
C6: KVATVQ C145A form 2
H41 Nε2
G143 N
A145 N
Q306 (P1)
H163 Nε2
F140 O
K301 (P6)
V305 (P2)
E166 Oε1
H164 O
E166 N
A303 (P4)
T304 (P3)
V302 (P5)
Q189 Nε2
Q189 Oε1
C7: NRATLQ C145A
H41 Nε2
G143 N
A145 N
Q306 (P1)
H163 Nε2
L305 (P2)
F140 O
Q189 Oε1
E166 Oε1
H164 O
T304 (P3)
E166 N
E166 O
A303 (P4)
T190 O
R302 (P5)
N301 (P6)
C8: SAVKLQ C145A
H41 Nε2
G143 N
A145 N
Q306 (P1)
H163 Nε2
N142 Oδ1
F140 O
Q189 Oε1
L305 (P2)
E166 Oε1
H164 O
E166 N
K304 (P3)
E166 O
V303 (P4)
T190 O
A302 (P5)
S301 (P6)
C9: ATVRLQ C145A
H41 Nε2
G143 N
A145 N
Q306 (P1)
F140 O
L305 (P2)
E141 O
H163 Nε2
Q189 Oε1
H172 Nε2
H164 O
R304 (P3)
E166 O
E166 N
V303 (P4)
T190 O
A301 (P6)
T302 (P5)

C10: REPMLQ C145A form 2

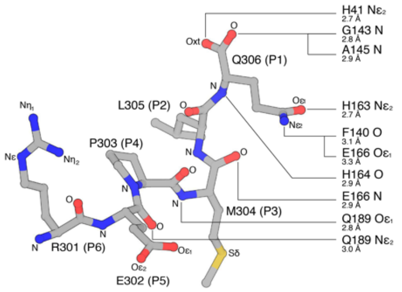

C12: PHTVLQ C145A

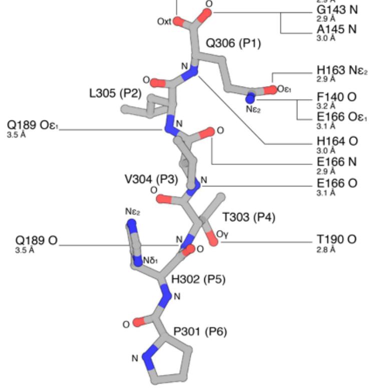

C12: PHTVLQ WT

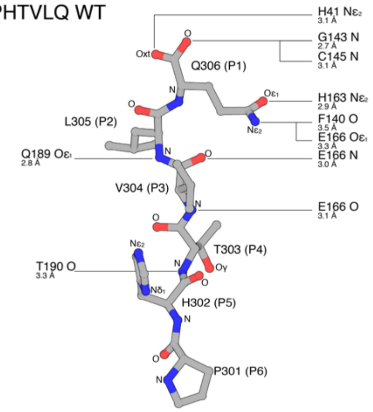

C12: PHTVLQ WT AE

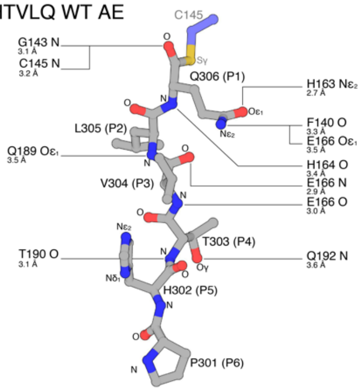

C13: NVATLQ C145A

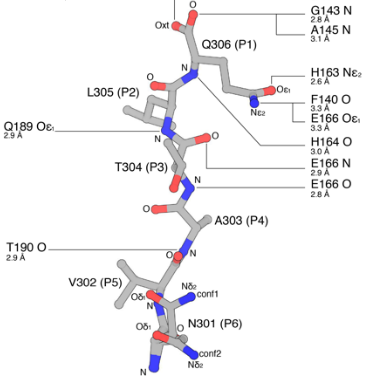

C13: NVATLQ WT form 2

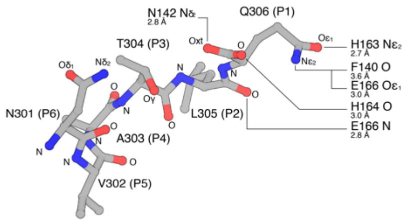

C14: TFTRLQ form 2

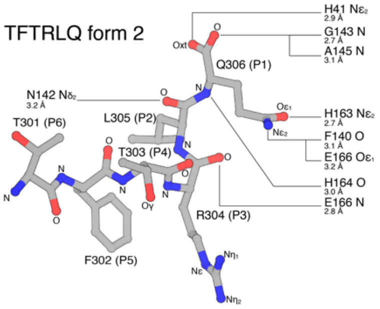

Supplementary Fig. 5. A summary of hydrogen bonding interactions between the P6 (301) through P1 (306) residue atoms of the 10 cleavage site variants and the atoms within the substrate binding site of M^{pro}. Standard deviations for the hydrogen bond lengths are listed for those interactions that occur more than once. Grey text signifies the atoms involving sidechain hydrogen bond interactions. Top left is a summary of all observed hydrogen bonds, side chain (light grey lines/values) or main chain (black lines/values). Black text signifies atoms involved in mainchain hydrogen bond interactions.

A

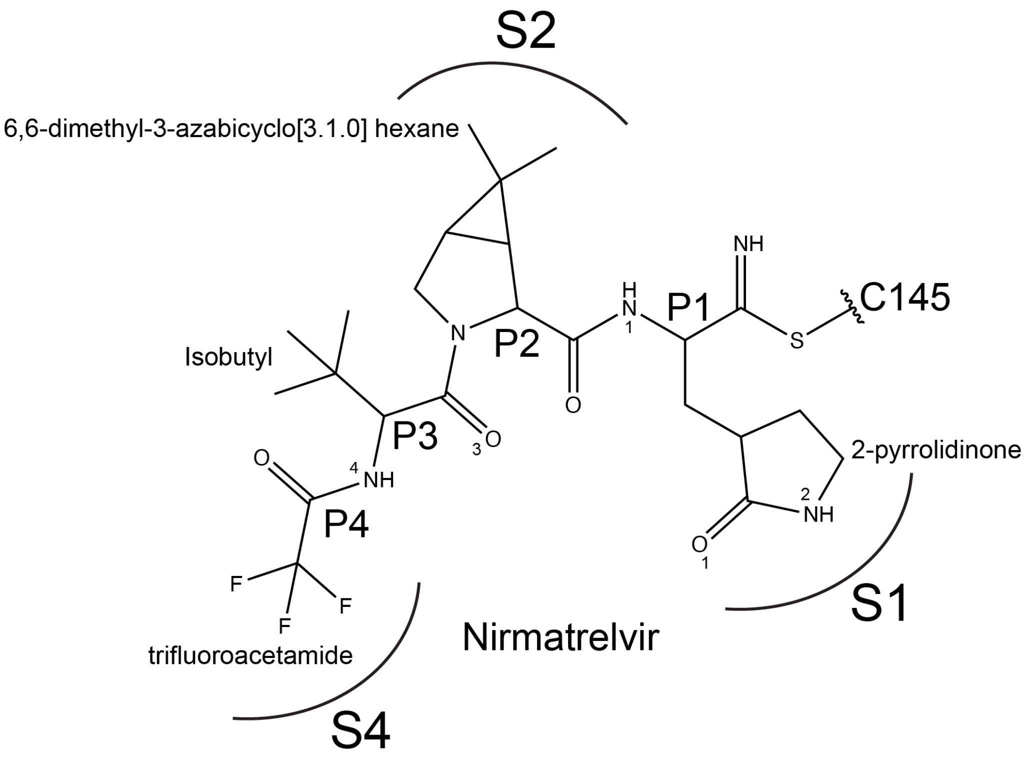

B

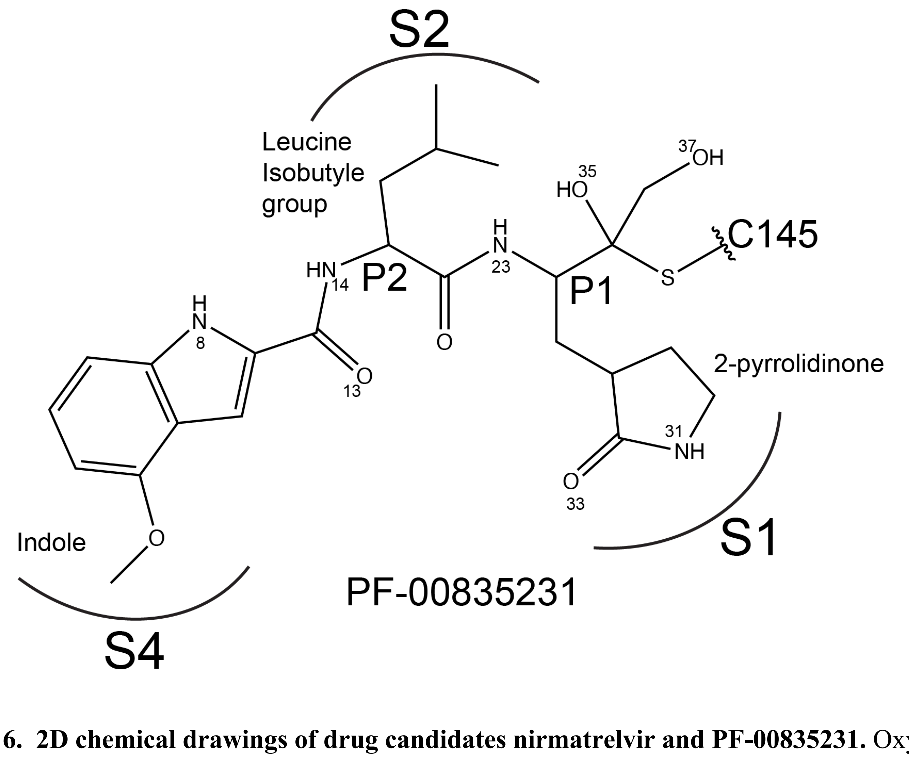

Supplementary Fig. 6. 2D chemical drawings of drug candidates nirmatrelvir and PF-00835231. Oxygen and nitrogen atoms involved in interactions in PDB 7RFS (A) or 6XHM (B) are numbered

Supplementary Table 1: Optimized M^{pro} gene and primer sequences used in this study

The pET28-SUMO-Mpro plasmid expresses His-SUMO-Mpro fusion protein

```
      -110       -100        -90        -80        -70        -60        -50
MGSSHHHHHH GSGLVPRGSA SMSDSEVNQE AKPEVKPEVK PETHINLKVS DGSSEIFFKI KKTTPLRRLM
       -40        -30        -20        -10         -1 1         10         20
EAFAKRQGKE MDSLRFLYDG IRIQADQTPE DLDMEDNDII EAHREQIGG- SGFRKMAFPS GKVEGCMVQV
        30         40         50         60         70         80         90
TCGTTTLNGL WLDDVVYCPR HVICTSEDML NPNYEDLLIR KSNHNFLVQA GNVQLRVIGH SMQNCVLKLK
       100        110        120        130        140        150        160
VDTANPKTPK YKFVRIQPGQ TFSVLACYNG SPSGVYQCAM RPNFTIKGSF LNGSCGSVGF NIDYDCVSFC
       170        180        190        200        210        220        230
YMHHMELPTG VHAGTDLEGN FYGPFVDRQT AQAAGTDTTI TVNVLAWLYA AVINGDRWFL NRFTTTLNDF
       240        250        260        270        280        290        300    306
NLVAMKYNYE PLTQDHVDIL GPLSAQTGIA VLDMCASLKE LLQNGMNGRT ILGSALLEDE FTPFDVVRQC SGVTFQ
```

Codon optimized M^{pro} gene sequence including N-terminal His_6-SUMO-tag

```
5’- CATCACCACCATCATCACGGCTCTGGATTGGTCCCCCGCGGCAGTGCTTCGATGAGCGACTCTGAA
GTTAATCAGGAGGCCAAACCTGAGGTAAAACCGGAAGTGAAGCCAGAGACTCATATTAACTTGAAGGTATCCGATGGCTCCTC
CGAGATTTTTTTCAAAATTAAAAAAACGACACCTCTGCGCCGTCTTATGGAGGCTTTCGCTAAACGTCAAGGAAAGGAAATGG
ACTCTCTGCGCTTTTTATATGATGGTATTCGTATTCAGGCCGATCAGACACCGGAAGACCTTGACATGGAAGATAACGACATC
ATTGAGGCCCACCGTGAACAGATCGGTGGGTCTGGCTTTCGTAAGATGGCCTTCCCATCAGGTAAAGTTGAGGGATGCATGGT
GCAGGTTACATGCGGCACTACGACGCTTAACGGCCTGTGGCTCGACGATGTGGTTTATTGCCCACGTCATGTGATTTGCACTT
CTGAAGACATGCTGAACCCAAATTATGAAGATTTACTGATTCGCAAAAGTAATCATAATTTTCTGGTACAGGCGGGGAACGTT
CAACTGCGCGTCATCGGGCACTCTATGCAGAATTGCGTCCTGAAGCTGAAAGTTGATACTGCGAACCCAAAAACACCAAAATA
TAAGTTTGTGCGCATTCAACCGGGCCAAACTTTCAGTGTTTTGGCTTGTTATAACGGCAGTCCGTCGGGTGTATATCAGTGCG
CAATGCGTCCTAATTTCACGATTAAGGGGTCTTTTCTCAATGGGTCCTGTGGTTCCGTTGGTTTTAATATTGACTATGATTGC
GTGTCATTCTGCTATATGCACCATATGGAGTTACCGACCGGAGTGCATGCCGGCACGGATCTGGAGGGCAATTTTTATGGCCC
TTTTGTAGATCGTCAGACCGCCCAAGCCGCTGGTACGGATACCACCATCACCGTGAATGTTTTAGCGTGGCTGTACGCAGCGG
TGATCAACGGCGACCGTTGGTTTTTGAATCGCTTTACTACAACGTTAAACGATTTCAACCTCGTTGCCATGAAGTACAATTAT
GAACCCCTCACTCAGGATCACGTCGACATCCTGGGTCCACTGTCGGCGCAGACAGGGATTGCCGTCCTGGATATGTGTGCGTC
ACTGAAAGAACTGTTGCAAAACGGGATGAACGGCCGTACAATCCTGGGTAGTGCGCTGCTGGAGGATGAGTTTACGCCGTTCG
ACGTGGTCCGGCAATGTAGTGGCGTGACCTTCCAATAA -3’
```

C4 – TSAVLQ		
	fwd	5’- CGTTCGACGTGGTCCGGCAATGTAGTGGCGTGACCTTCCAATAACTCGAGCACCACCACC -3’
	rev	5’- GGTGGTGGTGCTCGAGTTATTGGAAGGTCACGCCACTACATTGCCGGACCACGTCGAACG -3’
C5 – SGVTFQ		
	fwd	5’- GGTCCGGCAATGTACCTCAGCTGTTTTGCAATAACTCGAGCACCACCACCACCACCACTG -3’
	rev	5’- CAGTGGTGGTGGTGGTGGTGCTCGAGTTATTGCAAAACAGCTGAGGTACATTGCCGGACC -3’
C6 – KVATVQ		
	fwd	5’- CGTTCGACGTGGTCCGGCAATGTAAGGTAGCTACTGTGCAATAACTCGAGCACCACCACC -3’
	rev	5’- GGTGGTGGTGCTCGAGTTATTGCACAGTAGCTACCTTACATTGCCGGACCACGTCGAACG -3’
C7 – NRATLQ		
	fwd	5’- CGTTCGACGTGGTCCGGCAATGTAACCGAGCTACTTTGCAATAACTCGAGCACCACCACC -3’
	rev	5’- GGTGGTGGTGCTCGAGTTATTGCAAAGTAGCTCGGTTACATTGCCGGACCACGTCGAACG -3’
C8 – SAVKLQ		
	fwd	5’- CGTTCGACGTGGTCCGGCAATGTTCCGCAGTTAAGTTGCAATAACTCGAGCACCACCACC -3’
	rev	5’- GGTGGTGGTGCTCGAGTTATTGCAACTTAACTGCGGAACATTGCCGGACCACGTCGAACG -3’
C9 – ATVRLQ		
	fwd	5’- CGTTCGACGTGGTCCGGCAATGTGCCACAGTTCGTTTGCAATAACTCGAGCACCACCACC -3’
	rev	5’- GGTGGTGGTGCTCGAGTTATTGCAAACGAACTGTGGCACATTGCCGGACCACGTCGAACG -3’
C10 – REPMLQ		
	fwd	5’- CGTTCGACGTGGTCCGGCAATGTCGCGAACCTATGTTGCAATAACTCGAGCACCACCACC -3’
	rev	5’- GGTGGTGGTGCTCGAGTTATTGCAACATAGGTTCGCGACATTGCCGGACCACGTCGAACG -3’
C12 – PHTVLQ		
	fwd	5’- CGTTCGACGTGGTCCGGCAATGTCCCCATACTGTTTTGCAATAACTCGAGCACCACCACC -3’
	rev	5’- GGTGGTGGTGCTCGAGTTATTGCAAAACAGTATGGGGACATTGCCGGACCACGTCGAACG -3’
C13 – NVATLQ		
	fwd	5’- CGTTCGACGTGGTCCGGCAATGTAACGTAGCTACTTTGCAATAACTCGAGCACCACCACC -3’
	rev	5’- GGTGGTGGTGCTCGAGTTATTGCAAAGTAGCTACGTTACATTGCCGGACCACGTCGAACG -3’
C14 – TFTRLQ		
	fwd	5’- CGTTCGACGTGGTCCGGCAATGTACCTTCACTCGTTTGCAATAACTCGAGCACCACCACC -3’
	rev	5’- GGTGGTGGTGCTCGAGTTATTGCAAACGAGTGAAGGTACATTGCCGGACCACGTCGAACG -3’
C145A Substitution		
	fwd	5’- CGATTAAGGGGTCTTTTCTCAATGGGTCCGCTGGTTCCGTTGGTTTTAATATTGACTATG -3’
	rev	5’- CATAGTCAATATTAAAACCAACGGAACCAGCGGACCCATTGAGAAAAGACCCCTTAATCG -3’

Supplementary Table 2. Crystallization Conditions

Construct	C4<br>TSAVLQ<br>C145A	C6<br>KVATVQ<br>C145A (form 1)	C6<br>KVATVQ<br>C145A (form 2)	C7<br>NRATLQ<br>C145A	C8<br>SAVKLQ<br>C145A	C9<br>ATVRLQ<br>C145A	C10<br>REPMLQ<br>C145A	C12<br>PHTVLQ<br>C145A	C13<br>NVATLQ<br>C145A	C14<br>TFTRLQ<br>C145A	C12<br>PHTVLQ<br>WT	C13<br>NVATLQ<br>WT
Concentration (mg/mL)	9.4	14.8	14.8	11.6	9.0	25.8	20.3	15.9	12.4	16.0	25.2	13.5
Screen	PACT-D7‡	PACT-C8	JCSG-A9	JCSG-C4	PACT-H4‡	JCSG-D3	Class-B12	JCSG-A7	PACT-B3‡	PACT-B3	JCSG-G8	JCSG-F7
Crystal Condition	0.1M Tris (pH 8.0)<br>20% PEG 6K<br>0.3M NaCl	0.1M HEPES (pH 7.0)<br>20% PEG 6K<br>0.2M NH4Cl	(pH 6.3)<br>20% PEG 3350<br>0.2M NH4Cl	0.1M HEPES (pH 7.0)<br>10% PEG 6K	0.1M Tris (pH 8.5)<br>20% PEG 3350	0.1M Na/K phosphate (pH 6.2)<br>50% PEG200<br>0.2M NaCl	0.1M tri-Na Citrate (pH 5.6)<br>35% t-Butanol	0.9M CHES (pH 9.5)<br>20% PEG 8K	0.1M Na citrate (pH 5.6)<br>16% PEG 2K	0.1M MIB buffer (pH 6.0)<br>25% PEG 1500	0.15M DL-Malic acid (pH 7.0)<br>20% PEG 3350	0.8M Succinic Acid (pH 7.0)
Beamline	APS 23IDD	ALS bl502	APS 23IDB	CLS CMCF BM	APS 23IDB	APS 23IDB	CLS CMCF BM	APS 23IDB	ALS bl501	APS 23IDB	CLS CMCF BM	ALS bl502
Wavelength (Å)	1.033	0.979	1.033	1.521	1.033	1.033	1.180	1.033	0.977	1.033	1.180	0.979
Resolution	2.00 Å	1.80 Å	1.50 Å	2.31 Å	2.40 Å	2.67 Å	1.50 Å	2.49 Å	1.98 Å	2.20 Å	2.26 Å	1.60 Å
Space Group	$P22_12_1$	$P22_12_1$	$P2_1$	C2	$P2_12_12_1$	C2	$P2_1$	$P2_1$	C2	$P2_1$	$P2_1$	$P2_1$
Unit Cell Dimensions												
a, *b*, *c* (Å)	67.3,<br>107.4,<br>138.1	67.1,<br>108.5,<br>137.8	47.9,<br>105.1,<br>52.3	166.1,<br>174.9,<br>96.3	85.8,<br>117.2,<br>121.4	275.6,<br>217.2,<br>104.6	49.7,<br>107.4,<br>53.4	67.3,<br>107.9,<br>277.2	105.9,<br>216.8,<br>121.3	49.1,<br>105.5,<br>53.3	67.3,<br>107.9,<br>277.2	49.0,<br>107.4,<br>54.4
α, β, γ (°)	90.0,<br>90.0,<br>90.0	90.0,<br>90.0,<br>90.0	90.0,<br>104.5,<br>90.0	90.0,<br>106.3,<br>90.0	90.0,<br>90.0,<br>90.0	90.0,<br>111.0,<br>90.0	90.0,<br>103.8,<br>90.0	90.0,<br>90.7,<br>90.0	90.0,<br>94.0,<br>90.0	90.0,<br>104.0,<br>90.0	90.0,<br>90.7,<br>90.0	90.0,<br>103.5,<br>90.0
Chains in ASU	3 Chains	3 chains	2 chains	7 chains	4 chains	14 chains	2 chains	12 chains	7 chains	2 chains	12 chains	2 chains
Solvent content	49.1%	49.4%	33.5%	55.9%	44.5%	59.5%	38.8%	49.6%	57.4%	36.9%	52.5%	39.2%
Matthew's coefficient	2.41	2.43	1.85	2.79	2.22	3.03	2.01	2.44	2.88	1.95	2.59	2.02

‡ Crystal screen condition optimized.

Supplementary Table 3. X-ray Crystallographic Data Statistics

Construct	C4 TSAVLQ C145A	C5‡ SGVTFQ C145A	C6 KVATVQ C145A (form1)	C6 KVATVQ C145A (form 2)	C7‡ NRATLQ C145A	C8 SAVKLQ C145A	C9‡ ATVRLQ C145A	C10 REPMLQ C145A	C12‡ PHTVLQ C145A	C13 NVATLQ C145A	C14 TFTRLQ C145A	C12‡ PHTVLQ WT	C13 NVATLQ WT
**PDB Accession code**													
**Data collection**													
Space group	$P22_12_1$	$P2_1$	$P22_12_1$	$P2_1$	C2	$P2_12_12_1$	C2	$P2_1$	$P2_1$	C2	$P2_1$	$P2_1$	$P2_1$
Cell dimensions													
a, *b*, *c* (Å)	67.3, 107.4, 138.1	123.7, 80.3, 63.3	67.1, 108.5, 137.8	47.9, 105.1, 52.3	166.1, 174.9, 96.3	85.8, 117.2, 121.4	275.6, 217.2, 104.6	49.7, 107.4, 53.4	67.3, 107.9, 277.2	105.9, 216.8, 121.3	49.1, 105.5, 53.3	67.3, 107.9, 277.2	49.0, 107.4, 54.4
α, β, γ (°)	90.0, 90.0, 90.0	90.0, 90.2, 90.0	90.0, 90.0, 90.0	90.0, 104.5, 90.0	90.0, 106.3, 90.0	90.0, 90.0, 90.0	90.0, 111.0, 90.0	90.0, 103.8, 90.0	90.0, 90.7, 90.0	90.0, 94.0, 90.0	90.0, 104.0, 90.0	90.0, 90.7, 90.0	90.0, 103.5, 90.0
Resolution (Å)	37.98 - 2.00 (2.07 - 2.00)	26.16 - 2.00 (2.07- 2.00)	60.32 - 1.80 (1.86 - 1.80)	45.61 - 1.50 (1.55 - 1.50)	46.22 - 2.31 (2.53 - 2.31)	58.6 - 2.40 (2.49 - 2.40)	166.0 - 2.67 (3.02 - 2.67)	44.00 - 1.50 (1.55 - 1.50)	60.87 - 2.49 (2.58 - 2.49)	47.50 - 1.98 (2.05 - 1.98)	28.30 - 2.20 (2.28 - 2.20)	98.66 - 2.26 (2.50 - 2.26)	43.55 - 1.60 (1.65 - 1.60)
CC1/2	1.000 (0.514)	0.980 (0.619)	0.999 (0.624)	0.995 (0.954)	0.998 (0.566)	0.998 (0.751)	0.994 (0.690)	0.997 (0.628)	0.997 (0.628)	0.853 (0.405)	0.997 (0.483)	0.999 (0.591)	0.999 (0.854)
R_{sym} or R_{merge}	0.024 (0.700)	0.141 (0.885)	0.104 (1.705)	0.052 (0.251)	0.073 (0.734)	0.155 (1.139)	0.164 (1.027)	0.134 (1.212)	0.134 (1.212)	0.238 (1.823)	0.161 (2.005)	0.115 (1.157)	0.076 (0.777)
R_{pim}	0.024 (0.700)	0.085 (0.777)	0.043 (0.735)	0.026 (0.122)	0.046 (0.471)	0.045 (0.313)	0.068 (0.426)	0.054 (0.49))	0.054 (0.490)	0.099 (0.805)	0.065 (0.797)	0.048 (0.479)	0.030 (0.314)
$I / \sigma I$	13.29 (1.10)	7.71 (0.54)	14.10 (1.50)	21.52 (5.93)	11.20 (1.70)	14.29 (2.06)	8.50 (2.00)	18.68 (1.66)	9.10 (1.50)	11.65 (0.97)	9.64 (1.07)	10.7 (1.60)	17.12 (2.65)
Completeness (%)	99.92 (99.87)	96.03 (93.88)	99.95 (99.98)	94.74 (82.66)	93.10 (62.40)	99.83 (99.98)	92.20 (69.10)	94.96 (92.32)	72.6 (62.00)	99.90 (99.81)	99.82 (99.62)	93.3 0 (61.90)	96.94 (95.10)
Redundancy	2.0 (2.0)	2.8 (1.8)	12.0 (11.2)	5.1 (4.9)	3.4 (3.4)	12.6 (13.0)	6.8 (6.8)	6.9 (7.2)	7.0 (7.1)	6.2 (6.0)	7.0 (7.2)	6.4 (6.2)	7.1 (7.0)
**Refinement**													
Resolution	2.00 Å	2.00 Å	1.80 Å	1.50 Å	2.31 Å	2.40 Å	2.67 Å	1.50 Å	2.49 Å	1.98 Å	2.20 Å	2.26 Å	1.60 Å
No. reflections	68332 (6750)	40278 (3896)	93822 (9299)	75618 (6542)	80942 (4047)	48483 (4760)	120829 (15104)	82390 (7992)	100601 (5918)	188635 (18774)	26743 (2654)	115794 (5791)	69758 (6807)
R_{work} / R_{free}	0.206 / 0.235	0.227 / 0.261	0.179 / 0.217	0.154 / 0.182	0.190 / 0.237	0.177 / 0.239	0.202 / 0.237	0.162 / 0.191	0.194 / 0.264	0.176 / 0.223	0.192 / 0.245	0.197 / 0.248	0.154 / 0.186
No. atoms													
Protein	7087	4997	7635	5381	16871	9660	33657	5441	28308	18084	4793	28279	5327
Ligand/ion	2	0	0	0	168	10	613	1	14	191	17	40	67
Water	240	268	526	603	205	247	210	598	159	1394	115	286	469
B-factors (Å^2)													
Protein	67.6	34.3	49.6	21.9	64.0	53.8	68.3	27.7	59.5	45.9	54.6	54.0	25.3
Ligand/ion	62.0	0.00	44.4	32.3	71.0	55.5	72.3	30.5	50.8	63.3	61.9	54.3	34.1
Water	55.3	36.5	17.0	10.0	47.8	48.3	55.1	37.6	43.7	45.3	44.4	38.0	37.6
R.M.S. deviations													
Bond lengths (Å)	0.005	0.011	0.016	0.007	0.009	0.008	0.003	0.009	0.009	0.013	0.008	0.002	0.017
Bond angles (°)	0.78	1.59	1.39	0.95	1.24	0.99	0.57	1.18	1.10	1.27	1.01	0.52	1.55
Ramachandran													
favored (%)	97.51	97.04	97.46	98.52	95.58	97.77	97.29	97.85	94.56	97.54	96.22	97.95	97.67
allowed (%)	2.49	2.63	2.54	1.48	4.32	2.23	2.64	2.15	5.39	2.46	3.45	2.05	2.16
outliers (%)	0.00	0.33	0.00	0.00	0.09	0.00	0.07	0.00	0.06	0.00	0.33	0.00	0.17

† Previously reported

‡ Anisotropic cut-off applied to merged intensity data. See Table 4a and 4b.

Supplementary Table 4a.

"The anisotropic delta-B indicates the directional dependence of the intensity falloff with resolution and is defined as the difference between the two principal components with the most extreme values.
An anisotropic ΔB of 10 A^2 indicates mild anisotropy.
An anisotropic ΔB over 25 A^2 indicates strong anisotropy.
An anisotropic ΔB over 50 A^2 indicates severe anisotropy.
The stronger the anisotropy, the more beneficial it is to employ the ellipsoidal truncation and anisotropic scaling."

-UCLA-Doe Institute: Diffraction anisotropy server. UCLADOE Institute from https://srv.mbi.ucla.edu/Anisoscale/discussion

Structure	Uncorrected high resolution limit cut-off (Å)	Mean I / σ(I) in highest resolution shell	Completeness In highest resolution shell (%)	Estimates of resolution limits in reciprocal lattice directions, from mean I / σ(I) >1 (Å)			Anisotropic ΔB (Å²)	Resolution limit after STARANISO correction	Mean I / σ(I) In highest resolution shell after correction	Completeness (ellipsoidal) in highest resolution shell (%)
				h	k	l				
C145A										
C4 – TSAVLQ	2.00	1.6	100.0	1.99	2.28	1.89	13.0			
C5 – SGVTFQ*	2.00	0.6	93.6	2.00	2.01	2.00	7.1			
C6 – KVATVQ										
form 2	1.50	5.7	74.9	1.49	1.43	1.40	1.23			
form 1	1.80	1.5	100.0	1.66	1.98	1.66	11.4			
**C7 – NRATLQ**	**2.60**	**1.3**	**99.5**	**2.48**	**2.23**	**2.87**	**19.0**	**2.31**	**1.7**	**62.4**
C8 – SAVKLQ	2.40	1.0	99.6	2.14	2.25	2.31	7.80			
**C9 – ATVRLQ**	**2.80**	**0.8**	**99.8**	**3.22**	**2.81**	**2.67**	**39.9**	**2.67**	**2.0**	**69.1**
C10 – REPMLQ	1.50	1.5	92.0	1.55	1.50	1.50	0.74			
**C12 – PHTVLQ**	**2.60**	**0.7**	**99.7**	**2.51**	**3.08**	**2.71**	**25.4**	**2.49**	**1.5**	**62.0**
C13 – NVATLQ	1.90	1.0	99.9	2.21	1.92	1.95	5.01			
C14 – TFTRLQ	2.20	1.4	95.3	2.15	2.21	2.62	11.07			
WT										
**C12 – PHTVLQ**	**2.0**	**1.0**	**99.7**	**2.23**	**2.96**	**2.21**	**26.0**	**2.26**	**1.6**	**61.9**
C13 – NVATLQ	1.60	2.2	99.9	1.40	1.59	1.62	5.41			

*PDB ID: 7JOY was corrected with anisotropy server prior to deposition.

Tickle, I.J., Flensburg, C., Keller, P., Paciorek, W., Sharff, A., Vonrhein, C., Bricogne, G. (2018). STARANISO (http://staraniso.globalphasing.org/cgi-bin/staraniso.cgi). Cambridge, United Kingdom: Global Phasing Ltd.

Supplementary Table 4b.

	Diffraction limits (Å) and corresponding principal axes of the ellipsoid fitted to the diffraction cut-off surface as direction cosines in the orthogonal basis, and in terms of reciprocal unit-cell vectors:					Eigenvalues of overall anisotropy tensor on IFIs (Å²) and corresponding eigenvectors of the overall anisotropy tensor as direction cosines in the orthogonal basis, and in terms of reciprocal unit-cell vectors:				
	2.461	0.8468	0.0000	-0.5319	0.890 a* - 0.456 c*	51.93	0.8362,	0.0000,	-0.5484)	0.884 a* - 0.467 c*
**C7 – NRATLQ C145A**	2.311	0.0000	1.0000	0.0000	b*	44.22	0.0000,	1.0000,	0.0000)	b*
	2.882	0.5319	0.0000	0.8468	0.810 a* + 0.586 c*	79.33	0.5484,	0.0000,	0.8362)	0.825 a* + 0.565 c*
	2.674	0.9945	0.0000	0.1045	0.995 a* - 0.098 c*	62.97	0.9928	0.0000	0.1201	0.996 a* - 0.093 c*
**C9 – ATVRLQ C145A**	2.718	0.0000	1.0000	0.0000	b*	66.10	0.0000	1.0000	0.0000	b*
	3.268	-0.1045	0.0000	0.9945	-0.274 a* + 0.962 c*	113.43	-0.1201	0.0000	0.9928	-0.310 a* + 0.951 c*
	2.490	0.7106	0.0000	0.7035	0.241 a* + 0.970 c*	53.58	0.7131	0.0000	0.7010	0.243 a* + 0.970 c*
**C12 – PHTVLQ C145A**	3.039	0.0000	1.0000	0.0000	b*	88.68	0.0000	1.0000	0.0000	b*
	2.664	-0.7035	0.0000	0.7106	-0.231 a* + 0.973 c*	70.50	-0.7010	0.0000	0.7131	-0.229 a* + 0.973 c*
	2.490	0.7106	0.0000	0.7035	0.241 a* + 0.970 c*	53.58	0.7131	0.0000	0.7010	0.243 a* + 0.970 c*
**C12 – PHTVLQ WT**	3.039	0.0000	1.0000	0.0000	b*	88.68	0.0000	1.0000	0.0000	b*
	2.664	-0.7035	0.0000	0.7106	-0.231 a* + 0.973 c*	70.50	-0.7010	0.0000	0.7131	-0.229 a* + 0.973 c*

Supplementary Table 5. Chain Interactions

Construct	C4<br>TSAVLQ<br>C145A	C6<br>KVATVQ<br>C145A (form 1)	C6<br>KVATVQ<br>C145A (form 2)	C7<br>NRATLQ<br>C145A	C8<br>SAVKLQ<br>C145A	C9<br>ATVRLQ<br>C145A	C10<br>REPMLQ<br>C145A	C12<br>PHTVLQ<br>C145A	C13<br>NVATLQ<br>C145A	C14<br>TFTRLQ<br>C145A	C12<br>PHTVLQ<br>WT	C13<br>NVATLQ<br>WT
Resolution	2.00 Å	1.80 Å	1.50 Å	2.31 Å	2.40 Å	2.67 Å	1.50 Å	2.49 Å	1.98 Å	2.20 Å	2.26Å	1.60 Å
Space Group	$P22_12_1$	$P22_12_1$	$P2_1$	C2	$P2_12_12_1$	C2	$P2_1$	$P2_1$	C2	$P2_1$	$P2_1$	$P2_1$
Chains in ASU	3 Chains	3 chains	2 chains	7 chains	4 chains	14 chains	2 chains	12 chains	7 chains	2 chains	12 chains	3 chains
Representative Chains used for analysis	Chain C in active site of Chain B	Chain C in active site of Chain B	Chain A in active site of Chain A	Chain B in active site of Chain D	Chain B in active site of Chain A	Chain B in active site of Chain F	Chain A in active site of Chain A	Chain J in active site of Chain K	Chain B in active site of Chain F	Chain A in active site of Chain A	(Acyl)<br>C in active site of B<br>(Product)<br>D in active site of E	Chain A in active site of Chain A
Chain Interactions	A: truncated to 302<br>B: truncated to 302<br>**C: in active site of B**	A: truncated to 302<br>B: truncated to 302<br>**C: in active site of B**	**A: in active site of symm. Op. A**<br>**B: in active site of symm. Op. B**	**A: in active site of symm. Op. G**<br>**B: in active site of D**<br>**C: in active site of B**<br>**D: in active site of A**<br>**E: in active site of F**<br>**F: in active site of symm. Op. C**<br>**G: in active site of E**	**A: in active site of symm. Op. B**<br>**B: in active site of A**<br>C: truncated to 302<br>D: truncated to 303	**A: in active site of G**<br>**B: in active site of F**<br>**C: in active site of M**<br>**D: in active site of E**<br>**E: in active site of A**<br>**F: in active site of C**<br>**G: in active site of K**<br>**H: in active site of B**<br>**I: in active site of N**<br>**J: in active site of L**<br>**K: in active site of I**<br>**L: in active site of H**<br>**M: in active site of J**<br>**N: in active site of D**	**A: in active site of symm. Op. A**<br>**B: in active site of symm. Op. B**	A: truncated to 303<br>B: truncated to 303<br>**C: in active site of B**<br>**D: in active site of E**<br>E: truncated to 303<br>F: truncated to 302<br>G: truncated to 302<br>H: truncated to 302<br>**I: in active site of H**<br>**J: in active site of K**<br>K: truncated to 303<br>L: truncated to 302	**A: in active site of symm. Op. B**<br>**B: in active site of F**<br>**C: in active site of G**<br>**D: in active site of E**<br>**E: in active site of symm. Op. C**<br>**F: in active site of D**<br>**G: in active site of A**	**A: in active site of symm. Op. A**<br>**B: in active site of symm. Op. B**	A: truncated to 302<br>B: truncated to 302<br>**C: in active site of B**<br>**D: in active site of E**<br>E: truncated to 302<br>F: truncated to 302<br>G: truncated to 302<br>H: truncated to 302<br>**I: in active site of H**<br>**J: in active site of K**<br>K: truncated to 302<br>L: truncated to 302	**A: in active site of symm. Op. A**<br>**B: in active site of symm. Op. B**

Supplementary Table 6: P6-P1 buried surface area

Construct	Buried Surface Area by M^{pro} Catalytic Site Position (Å^2)						Total
	P6	P5	P4	P3	P2	P1	
C4 – TSAVLQ	43	53	102	45	168	213	624
C5 – SGVTFQ	53	30	66	30	128	146	452
C6 – KVATVQ	39	68	101	44	134	215	601
C6 – KVATVQ (form 2)	34	12	16	93	114	212	481
C7 – NRATLQ	32	73	100	50	165	212	632
C8 – SAVKLQ	49	40	133	70	144	207	643
C9 – AVTRLQ	0	73	130	77	163	219	662
C10 – REPMLQ (form 2)	71	14	54	165	159	218	681
C12 – PHTVLQ	41	56	127	51	158	204	637
C13 – NVATLQ	40	63	94	51	163	215	625
C14 – TFTRLQ (form 2)	15	68	25	109	122	209	548

Supplementary Table 7. Summary of commonly observed P6-P1 hydrogen bond interactions

M^{pro} Catalytic Site Position	Hydrogen Bond	Occurrence	Mean Distance	Max Distance	Min Distance	Mean Bond Angle
P4	303[N] - T190[O]	6	2.9 ± 0.1 Å	3.1 Å	2.7 Å	167° ± 6°
P3	304[N] - E166[O]	8	2.9 ± 0.1 Å	3.2 Å	2.8 Å	162° ± 7°
	304[O] - E166[N]	11	2.9 ± 0.1 Å	3.0 Å	2.8 Å	161° ± 3°
P2	305[N] - Q189[OE1]	7	2.9 ± 0.1 Å	3.0 Å	2.7 Å	155° ± 6°
P1	306[N] - H164[O]	11	3.1 ± 0.2 Å	3.5 Å	2.9 Å	167° ± 7°
	306[O] - A145[N]	10	3.0 ± 0.1 Å	3.3 Å	2.8 Å	170° ± 6°
	306[O] - G143[N]	11	2.8 ± 0.1 Å	2.9 Å	2.7 Å	139° ± 4°
	306[OXT] - H41[NE2]	10	2.8 ± 0.1 Å	3.0 Å	2.7 Å	168° ± 7°
	306[NE2] - E166[OE1]	10	3.3 ± 0.1 Å	3.4 Å	3.1 Å	130° ± 4°
	306[NE2] - F140[O]	11	3.1 ± 0.1 Å	3.3 Å	3.0 Å	141° ± 10°
	306[OE1] - H163[NE2]	11	2.7 ± 0.1 Å	2.9 Å	2.6 Å	166° ± 12°
